# Using Deep Graph Neural Networks Improves Physics-Based Hydration Free Energy Predictions Even for Molecules Outside of the Training Set Distribution

**DOI:** 10.1021/acs.jpcb.5c02263

**Published:** 2025-07-11

**Authors:** Luke H. Elder, Alexey V. Onufriev

**Affiliations:** † Department of Computer Science, Virginia Tech, Blacksburg, Virginia 24061, United States; ‡ Department of Physics, Virginia Tech, Blacksburg, Virginia 24061, United States; § Center for Soft Matter and Biological Physics, Virginia Tech, Blacksburg, Virginia 24061, United States

## Abstract

The accuracy of computational water models is crucial to atomistic simulations of biomolecules. Here we explore a decoupled framework that combines classical physics-based models with deep neural networks (DNNs) to correct residual error in hydration free energy (HFE) prediction. Our main goal is to evaluate this framework on out-of-distribution data (molecules that differ significantly from those used in training), where DNNs are known to struggle. Several common physics-based solvation models are used in the evaluation. Graph neural network architectures are tested for their ability to generalize using multiple data set splits, including out-of-distribution HFEs and unseen molecular scaffolds. Our most important finding is that for out-of-distribution data, where DNNs alone often struggle, the physics + DNN models consistently improve physics model predictions. For in-distribution data, the DNN corrections significantly improve the accuracy of physics-based models, with a final RMSE below 1 kcal/mol and a relative improvement between 40% and 65% in most cases. The accuracy of physics + DNN models tends to improve when the 6% of molecules with the highest experimental uncertainty are removed. This study provides insights into the potential and limitations of combining physics and machine learning for molecular modeling, offering a practical and generalizable strategy of using DNN as independent postprocessing correction.

## Graphical abstract

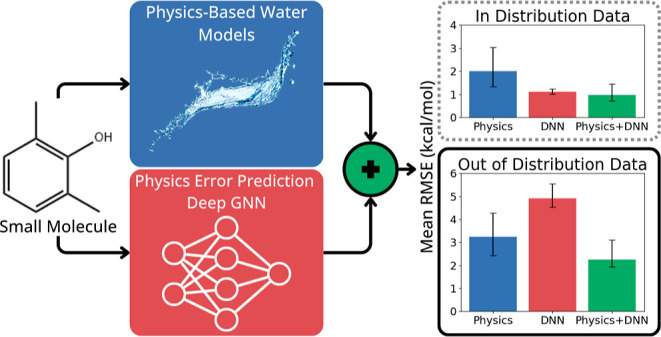

## 1 Introduction

Atomistic modeling and simulation methods provide a powerful framework for biological research,^1–4^ forming the foundation for modern approaches to structure-based drug design.^5^ The ability of these methods^6^ to address biologically relevant problems is largely determined by how accurately and computationally efficiently they treat the complex solvation and electrostatic effects in biomolecules surrounded by water. As a result, a wide range of water models have been developed over the years, each lacking perfection^7–10^ and striking a different compromise between speed and accuracy; see, e.g., ref 11 for a recent review. The significant limitations of current models underscore the ongoing need for improved methods to capture solvent effects accurately in biomolecular simulations.

In general, two main strategies, each with its own advantages and limitations, are typically employed for atomistic modeling of aqueous solvation: explicit and implicit.^11^ In explicit solvation, each water molecule is represented at the same atomic resolution as the biomolecule, enabling detailed modeling of atomic interactions at the cost of large computational expense.^12–19^ In contrast, implicit solvation methods approximate the solvent as a continuous dielectric medium, eliminating the need to explicitly simulate every water molecule.^20–30^ Among these, the generalized Born (GB) approximation^25,31–63^ is popular due to its balance between computational efficiency^64^ and its reasonably accurate representation of electrostatic solvation effects, but it also has several limitations.^65^ None of these methods, explicit or implicit, fully capture all aspects of the complex behavior of water, including quantum-mechanical effects. Although fully quantum models hold the promise of a more fundamental description,^66^ they remain prohibitively expensive for large systems on biologically relevant time scales. Various approximations to full quantum reality become inevitable, and, consequently, even the most advanced approaches that are capable of practical simulations still exhibit stubborn residual errors.

Machine learning (ML) approaches,^67,68^ including deep neural networks (DNNs),^69–71^ have become widely used and have already made significant impacts in the field of computational chemistry.^72–87^ Graph neural networks (GNNs) are a popular deep learning approach due to their invariance to molecular symmetries.^88–90^ Several ML works in this field focus on strategies designed to improve the accuracy of the description of solvation effects.^91–99^

Despite some impressive results, the overall success of state-of-the-art black-box ML models in science has been limited due to data set constraints and difficulties in producing physically consistent predictions on out-of-distribution data.^100,101^ Given the limitations of both physics and ML-based methodologies, physics-guided machine learning (PGML)^102–104^ has emerged as an area of research that aims to utilize physics knowledge in the design and training of ML models to achieve better generalization accuracy on samples outside of training data. One PGML research direction that has received considerable attention is focused on incorporating various physics-based constraints directly in the process of training ML models to provide additional sources of supervision to ML models beyond the empirical loss observed on labeled training data. This direction has been explored in several scientific applications including protein–ligand binding,^73,105,106^ lake modeling,^107,108^ quantum mechanics,^109^ and solving partial differential equations.^110,111^ For prediction of hydration free energies (HFEs) in conjunction with physics-based modeling, ML has been used to train the parameters of the physics-based model, e.g., the force-field.^93^

Another common PGML approach designed to directly address the inaccuracies of physics-based models is residual modeling, where an ML model learns to predict the error of physics-based predictions. Traditionally, this approach has used simple ML models, such as linear regression^112,113^ or kernel ridge regression,^85^ to learn the residual function in an effort to minimize overall systematic error. Recently, more expressive models such as random forests and gradient-boosted regression have been used to learn corrections to classical protein–ligand binding affinity scoring functions with impressive results.^114–116^ DNNs have also been used for residual modeling to solve differential equations^117,118^ and predict extreme weather events.^119^

In an earlier work,^98^ we proposed a strategy to harness the power of DNNs to learn the residual error of physics-based HFE predictions for small molecules. Specifically, deep GNNs were used as an independent postprocessing step to correct the HFE prediction errors of several classical physics-based water models. The motivation for this work was several-fold. First, accurate prediction of HFE is important in its own right:^120^ this single number incorporates many aspects of the complex physics of hydration. Having the ability to predict HFE correctly is key to many types of computations common to molecular biophysics and computational biology,^121^ including prediction of receptor–ligand binding energetics.^122–126^ The accuracy of HFE predictions is sensitive to the accuracy of the underlying water model at ambient conditions; the quality of the latter is paramount to success of modeling and simulation efforts in many areas,^127^ including atomistic simulations of biomolecules. It is also important that a reasonably large data set of experimental HFEs is available,^128^ allowing the DNN to be trained directly against the experiment, rather than against yet another model. Second, by using the strategy of residual modeling, the physics-based model generates the largest component of the final prediction by handling the known physics, which it accurately described. The relatively small inaccuracies that remain are complex, hard to decompose into distinct independent components, and generally difficult or even impossible to describe accurately within computationally facile models of classical physics. A GNN, which is invariant to molecular symmetries, is effective at learning complex patterns and thus well suited for the task of learning these convoluted residuals. Finally, a potential practical benefit of this strategy is that it is an easy way to enhance the accuracy of any physics-based model that predicts the quantity of interest, with no additional, and often nontrivial, effort to integrate the physics-based and ML parts. Bass et al.^98^ demonstrated that DNN postprocessing corrections to the physics-based HFE predictions consistently reduced the error to experiment on unseen test data. These findings suggest that this approach has potential for practical use within the field.

A key question that remains unanswered is the performance of the DNN postprocessing approach on out-of-distribution data, that is, data that are not only unseen, but also dissimilar to what was used for training. Overfitting of DNN models to the training set distribution is a well-known problem that goes beyond prediction of HFEs; that general problem persists despite recent progress within the field. This leads to the question of how well DNN models within this field would generalize to out-of-distribution data. To address this issue, here we explore how the DNN postprocessing approach generalizes to out-of-distribution data in the context of HFE prediction, due to its importance and computational facility. To this end, we carefully create two splits of the HFE data set specifically designed to demonstrate performance on out-of-distribution data. In addition, we utilize two well-established GNN models and test multiple feature sets. The overall goal of this work is to better understand the limitations and safety of using the DNN postprocessing approach to generate HFE predictions even for out-of-distribution samples.

In this work, we choose to employ GNNs rather than other non-DNN machine learning approaches such as gradient boosted regression trees (GBRT) for several reasons. First, while non-DNN approaches have been quite successful in predicting HFEs,^97,99,129–131^ deep learning approaches have also seen success in this area, even with small data sets.^129,132,133^ For example, Wu et al.^129^ showed that GNN-based methods consistently outperformed non-DNN methods on the FreeSolv data set.^128^ Due to their impressive performance, it is well-known that DNNs are becoming more and more prevalent within the field. Given their important role in the field, it is our goal to test our hybrid physics + machine learning approach, and its generalization, with a DNN, not necessarily to choose the absolute best model for this task. To that end, if the DNN postprocessing approach generalizes well with our small data set, this will indicate that DNNs are well suited for this approach even when experimental data are very limited. Finally, using a GNN specifically gives us more freedom in the types of features we can include. Methods such as GBRT require a single feature vector as input, which leads to limitations, while using a GNN with molecular graphs as input allows for directly including structure-based features such as distances between atoms and partial charges. To improve the robustness and generalization of our conclusions, in this work we also train GBRT models within our approach, and compare these results with those from the GNNs.

The remainder of this work has the following structure. The extensive “Methods” section begins with an introduction to the overall approach, then describes the choice of physics-based solvent (water) models used here; their details and parameters are given in the Supporting Information. The “Methods” section proceeds to define and describe the neural networks used, the featurization, and the data set with different data set splits. The accuracy of our approach on the test set for each split of the data set is shown in “Results and Discussion.” The overall findings are summarized and discussed further in “Conclusions,” along with the limitations of the approach.

## 2 Methods

Some residual error to experiment exists for HFE predictions for every practical physics-based model. Here, we train a deep graph neural network to predict this error. To do this, we minimize the mean square error (MSE) between the physics prediction plus the DNN predicted error and experiment. The overall strategy can be seen in Figure 1 and the loss function is defined as follows

1 $$L_{mse}=\frac{\sum_{i=1}^{N}{\left(y_{expt,i}-\left(y_{phys,i}+y_{gnn,i}\right)\right)}^{2}}{N}={RMSE}^{2}$$

where *N* is the number of data points in a batch, *y*_expt,*i*_ are the experimental HFE values, *y*_phys,*i*_ are the predicted values of the physics model, *y*_gnn,*i*_ are the predicted corrections from the deep graph neural network, and RMSE is the root-mean-square error for the final predictions relative to the experimental values. While other equivalent forms of the loss function exist, we have chosen this form to ensure consistency with Figure 1.

**Figure 1:**
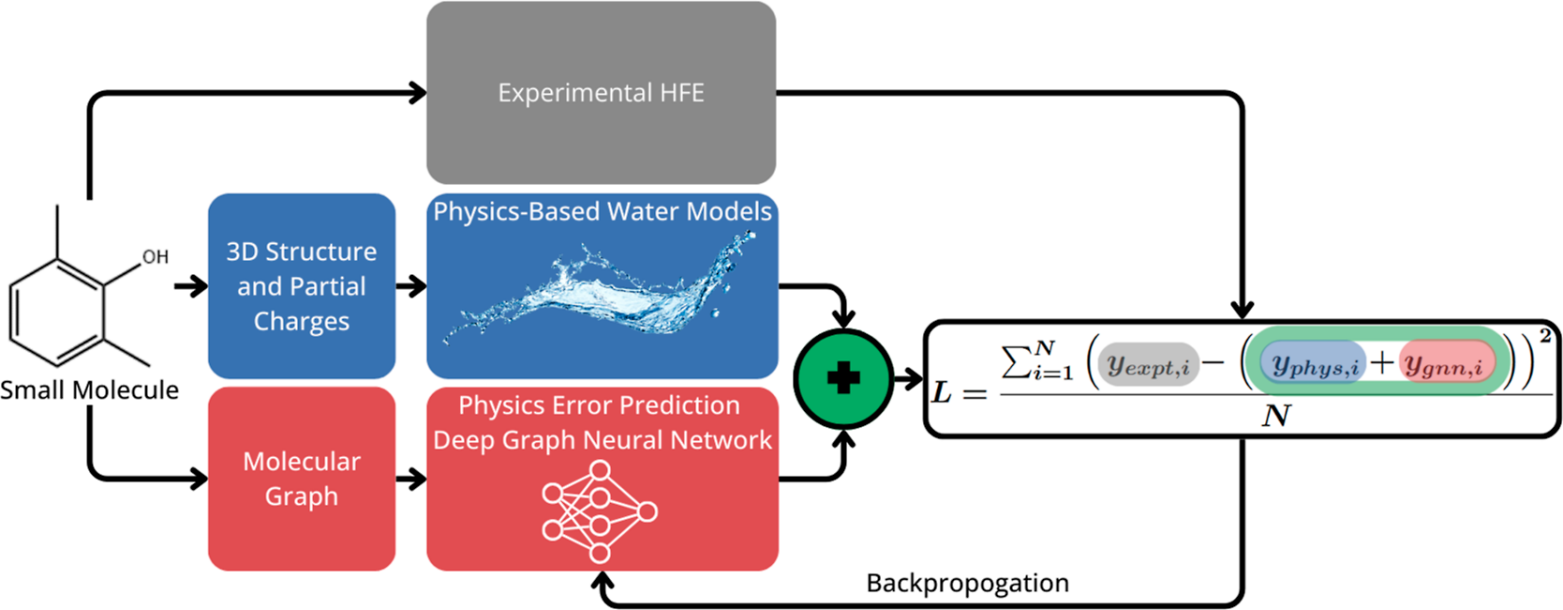
Schematic showing our overall approach of using DNN to reduce the remaining error between the hydration energies predicted by physics-based models and experiment. The DNN is trained to minimize the remaining error of the “physics + DNN” prediction. The physics and the DNN parts of the overall workflow are completely separate and independent: the output of the physics-based model is only used in the loss for the DNN.

### 2.1 Physics-Based Water Models

The five classical water models used here as baselines for evaluating ML improvements include one explicit model, TIP3P, and four implicit models: CHA-GB, GBNSR6, AASC, and IGB5 (GB-OBC), listed in approximate order of expected accuracy. GBNSR6 is used with two different radii sets: mbondi and ZAP9.^120^ The former is a general use radii set for solvation models while the latter has been optimized specifically for small molecule HFE prediction. Further details about these models and their parameters can be found in the Supporting Information.

The main rationale for selecting this particular subset from the many available classical solvent models is to assess our new approach across a broad range of accuracy/speed trade-offs characteristic of solvation models currently used in practice (see Table 1). A great variety of physics-based water models developed for atomistic simulations is available, yet none of the current models commonly used in practice is perfect:^7–10^ various compromises between speed and accuracy has to be made, see, e.g., ref 11 for a recent review. All of the water models considered in this work are classical, making a hardly avoidable compromise between computational efficiency and accuracy: the models account for quantum effects only in an average sense, through their (fixed) parameters. Further approximations depend on the specific water model: for example, implicit solvent models replace the discrete water molecules with a structureless continuum. Although the implicit models alone span a wide range of trade-offs, we include an explicit model to ensure that our conclusions are robust to the type of solvation model. The chosen implicit models illustrate two aspects of the evolution of the framework: first, improvements in accuracy while maintaining the same physical assumptions (e.g., IGB5­(GB-OBC) to GBNSR6), and second, gains achieved by introducing additional physical insights absent in earlier approximations (e.g., GB to CHA-GB). Specifically, CHA-GB^134^ is a generalized Born model modified to account for charge hydration asymmetry (CHA)—the noninvariance of the polar solvation energy with respect to solute charge inversion. While IGB5 (GB-OBC) and AASC are used with a simple, single-parameter model for the nonpolar component of the free energy, a more advanced treatment of the nonpolar term is applied with GBNSR6 and CHA-GB; see Supporting Information for details. Our selection of TIP3P as the sole representative of explicit solvent models is motivated by several considerations. First, based on the limited published data available,^13^ fixed-charge explicit models such as TIP3P, though widely used and relatively fast, do not offer the same breadth of HFE accuracy variation as the implicit models. Thus, including a single representative is sufficient for our purposes. Although other water models, including special-purpose models,^135,136^ can outperform TIP3P in reproducing certain water properties, see ref 11 for a review, it remains unclear^137^ whether they yield meaningful improvements in our primary accuracy metric: the RMSE of small-molecule HFE predictions compared to experiment. Moreover, more accurate water models, such as polarizable ones, would make the computation of HFEs for a large enough data set much more computationally expensive than is already the case with TIP3P, Table 1. Since our goal is to evaluate our general strategy, rather than evaluating specific water models, we believe that limiting the representative examples to the above five models is appropriate. It is worth noting that we intentionally exclude the broad class of quantum mechanical (QM)-based HFE models from consideration; see, e.g., ref 138 for a comprehensive review. The reason for the exclusion of QM-based approaches due to two primary reasons: most importantly, unlike classical models of solvation, which necessarily miss some physics of the process, quantum mechanics is capable of predicting molecular properties essentially exactly in principle. Additionally, from a practical perspective, QM-based models typically fall into a different class of computational complexity, especially when compared with fast, classical implicit solvent models.

**Table 1:** Accuracy-Efficiency Range Offered by the Physics-Based Water Models Considered in This Work^a^

model	RMSE	approximate simulation time
TIP3P	1.54	2 day/molecule^139^
AASC	2.51	100 ms/molecule
CHA-GB	1.72	30 ms/molecule
GBNSR6 (ZAP9)	1.67	30 ms/molecule
GBNSR6 (mbondi)	2.25	30 ms/molecule
IGB5	2.84	5 ms/molecule

a Shown are the RMSE (kcal/mol) relative to the experiment and approximate simulation time (for TIP3P model see ref 139) to obtain a converged estimate of the HFE of a small molecule using one CPU core for each of the physics-based water models (ms = milisecond). The numbers are averages over the entire FreeSolv database.

### 2.2 Deep Neural Networks

We used two different deep graph neural networks to test our approach. We use a model implementing the graph convolutions from Duvenaud et al.^89^ which is the model used in our previous work.^98^ We also use a message passing neural network (MPNN) based on Gilmer et al.^88^ We intentionally use two simple but highly tested models because of our desire to evaluate our overall strategy and its generalization rather than to maximize the accuracy of a single model. In both cases, we use implementations in DeepChem.^70^ Details for the architectures of each models are seen below. We also compare these results against those for GBRT. Details for the GBRT models are in the Supporting Information.

#### 2.2.1 GraphConv

The GraphConvModel, implemented in TensorFlow^140^ by DeepChem^70^ and based on the graph convolutions described by Duvenaud et al.,^89^ is used to predict the difference between the experimental values and the predictions of the physics models. As input, the model takes graph representations of molecules, with atoms as nodes and bonds as edges. Details of the featurization can be found in Section 2.3.

The model itself consists of two convolutional layers and an atom-level linear layer with ReLU activation used for each layer. The convolutional layers are size 53 and 38, respectively, while the atom-level linear layer is size 27 as optimized in Bass et al.^98^ Dropout of 0.4 is used after all layers during training as a regularization tool to improve the robustness of the model while training.^141^ Each convolutional layer aggregates information from neighboring nodes. After this, graph pooling is applied, which decreases the size of the graph representation while maintaining the most important information. The output from the second graph pool layer is used as input to the atom-level linear layer, which transforms each node representation. The graph gather takes the output of the linear layer and combines the information from the graph to a fixed-size representation. This representation is then used as input for the final linear layer that transforms the data to give its final prediction.

#### 2.2.2 MPNN

The MPNN model is implemented as a TorchModel in DeepChem^70^ based on the MPNN framework proposed by Gilmer et al.^88^ The model itself is created using the Deep Graph Library^142^ and PyTorch^143^ and is used to predict the difference between experimental values and predictions of the physics models. As input, the model takes graph representations of molecules, with atoms as nodes and bonds as edges. Details of the featurization can be found in Section 2.3.

The model consists of two main components: the message passing stage and the readout and prediction stage. The message passing stage begins by linearly transforming the input node features to the node embedding size, which is set to the default value of 64. The model then iterates over 3 steps of message passing. Each message passing step begins with message passing using the NNConv layer in the Deep Graph Library^142^ where information is passed between neighboring nodes conditioned by the edge features. The number of hidden edge features is set to the default of 128. This is followed by ReLU activation and a gated recurrent unit. After this, a Set2Set^144^ layer using 6 Set2Set steps is used to aggregate the from all nodes to create a fixed length graph representation. Two linear layers are used to transform this output into a final prediction.

Described very simply, the Set2Set layer uses a long short-term memory (LSTM) network to learn how to combine the node features of a graph into a single fixed-length representation. This approach is commonly used and has been shown to result in more expressive models compared to simpler approaches such as taking the sum or mean of node features.^88^

### 2.3 Featurization

#### 2.3.1 Construction of Graphs

Individual graphs are used to represent each molecule as input to the graph neural networks. Each heavy atom is represented by a node and each bond by an edge. Hydrogen atoms do not have their own node and are only implicitly included. All results presented use this overall strategy. Before arriving at this final featurization scheme, we tested other strategies including explicit hydrogen atoms and including edges between nonbonded atoms. However, preliminary results indicated that there was no overall improvement in accuracy from these alternative approaches. A feature vector is created for each node and edge to describe the atoms and bonds in the graph. Categorical features were one-hot encoded while numerical features were min–max normalized to be between 0 and 1.

#### 2.3.2 Selection of Features

The final features used were selected from two sets of features: chemistry-based features and physics-based features. We define chemistry-based features as those that can be easily determined by knowing the molecular structure, while physics-based features are those that require physics-based calculations to compute. Given the limited size of our data set (see Section 2.4), we wanted a relatively small feature set. The rationale for selecting the final features used is presented below. The final feature sets are shown in Table 2.

**Table 2:** Summary of All Features Used^a^

		feature name	type	size	set of values
node features	chemistry-based	atom identity	categorical	9	C, N, O, F, P, S, Cl, Br, I
		atom degree	categorical	6	0–5
		attached H	categorical	5	0–4
		hybridization	categorical	3	sp, sp^2^, sp^3^
		aromaticity	binary	1	
	physics-based	partial charge	numerical	1	
		inverse Born radius	numerical	1	
				
edge features	chemistry-based	bond type	categorical	4	single, double, triple, aromatic
	physics-based	inverse distance	numerical	1	

a Type indicates whether the feature is categorical, binary, or numerical. The value in the size column indicates how many spaces the feature takes up on the feature vector. Set of values indicates all values that were considered for categorical features.

The following chemistry-based node features were initially considered: atom identity, atom degree (number of attached heavy atoms), number of attached hydrogens, hybridization (sp, sp^2^, or sp^3^), aromaticity, integer charge, hydrogen bond donor or acceptor, and electronegativity. Integer charge and hydrogen bonding donor or acceptor information were excluded because they were very sparse with very few atoms having nonzero values for these features, meaning that the model may have difficulty learning how they affect things. Electronegativity was excluded because it is determined entirely by atom identity meaning that having both features would contain redundant information. The following chemistry-based edge features were considered: bond type, both atoms in the same ring (binary), conjugation, and stereo configuration. Only bond type was kept because the stereo configuration was very sparse and the other two features were highly correlated with bond type.

The physics-based node features initially considered were partial charge and two features calculated using GBNSR6:^63,145^ inverse effective Born radius and the atom’s individual contribution to the polar solvation energy calculated as Δ*G*_*ij*_^pol^ where *i* = *j* from the generalized Born equation

2 $$\Delta G_{pol}=\sum_{ij}\Delta G_{ij}^{pol}\approx -\frac{1}{2}\left(\frac{1}{\epsilon_{in}}-\frac{1}{\epsilon_{out}}\right)\frac{1}{1+\beta \alpha }\sum_{ij}q_{i}q_{j}\left(\frac{1}{f_{ij}^{GB}}+\frac{\alpha \beta }{A}\right)$$

See the Supporting Information for more details about GBNSR6. Inverse distance between nodes and the pairwise contribution to the solvation energy, Δ*G*_*ij*_^pol^ where *i* ≠ *j*, were initially considered as physics-based edge features. The solvation energy contributions, Δ*G*_*ij*_^pol^, for both the nodes (*i* = *j*) and the edges (*i* ≠ *j*) were excluded from the final featurizations due to their dependence on other features. Thus, the final physics-based features used are the partial charge and the inverse Born radius for nodes and the inverse distance for edges only.

#### 2.3.3 Final Featurizations

For the GraphConvModel, we used the chemistry-based node features, which is very similar to what was used in Bass et al.^98^ Note that edge features are not used in this model because its convolutions do not consider edge information. For the MPNN, we tested three different feature combinations: chemistry-based features, physics-based features, and all features from both sets. The models trained with these different feature sets are referred to as “MPNN (chem)”, “MPNN (phys)”, and “MPNN (all)”, respectively, in the tables and figures below.

### 2.4 Data Set

The DNN models are trained and evaluated using version 0.52 of the FreeSolv database^128^ which is found at the following URL: https://github.com/MobleyLab/FreeSolv. This database, described in Table 3, is a collection of experimental HFEs for 642 neutral small molecules.

**Table 3:** FreeSolv Database Version 0.52: Experimental Hydration Free Energies for Small Neutral Molecules^a^

	full data set	low-uncertainty subset
number of molecules	642	602
mean number of atoms (non-H)	8.7 ± 4.2	8.3 ± 3.7
number of atoms (non-H) range	(1, 24)	(2, 23)
mean HFE, μ ± σ	–3.80 ± 3.85 kcal/mol	–3.67 ± 3.83 kcal/mol
HFE range	(−25.47, 3.43) kcal/mol	(−25.47, 3.43) kcal/mol
mean uncertainty, μ ± σ	0.57 ± 0.31 kcal/mol	0.51 ± 0.18 kcal/mol
uncertainty range	(0.03, 1.93) kcal/mol	(0.03, 1.22) kcal/mol
elements (most to least frequent)	C, O, Cl, N, F, S, Br, P, I	C, O, Cl, N, F, S, Br, I, P

a The low-uncertainty subset column shows the summary information after excluding molecules with the highest uncertainty (see Section 2.4.3 below for details) and with only a single heavy atom.

The FreeSolv data set was selected over the larger MNSol^146^ and CompSol^147^ data sets for several reasons. The additional data points in these other data sets come primarily from solvation data for nonaqueous solvents and at nonambient temperatures. Our method relies on physics-based solvation models that have been optimized to model water at ambient conditions, which is of primary interest. Potential adaptation of these models to other solvents/conditions is nontrivial, would likely include major reparameterization efforts that would be necessary to ensure reasonably accurate physics-based predictions for different solvents and temperatures, which is beyond the scope of this work. Thus, our method is restricted to HFE predictions at ambient conditions, which, we argue, is most relevant to biomolecular simulations. We focus on neutral solutes to avoid known complications related to the measurement and prediction of HFEs of charged species.^148,149^ For neutral solutes at ambient conditions, the FreeSolv data set contains the largest number of data points at 642. The MNSol data set contains 541 HFEs for neutral small molecules, while the CompSol data set contains HFEs for 581 unique solutes but not all of them are at ambient conditions. Additionally, the FreeSolv data set provides experimental uncertainties of each HFE allowing us to filter out high uncertainty values and analyze their impact on model performance.

#### 2.4.1 Data Splitting into the Training and Test Sets

Three different data splits were tested, each using a 6:1:1 ratio of data for training, validation, and test sets. The first tests how well the models can make predictions on test data similar to training data (test data is in the training set distribution). This data split is identical to what was used in Bass et al.^98^ The data set was separated into test and validation sets of 80 molecules each, and the remaining 482 molecules were used as the train set. These sets were selected using stratified sampling, each group representing a different range of HFEs, to ensure that each partition had a similar distribution of HFEs as the whole data set. For more details, see the Supporting Information of Bass et al.^98^

The second data split is used to test how the models generalize to molecules with HFE outside the range of those seen in training data. The 80 molecules with the highest absolute HFE are selected as the test set. The validation set of 80 molecules is separated from the training set of 482 using the same stratified sampling technique used for the first data split. Thus, the training and validation sets have the same distribution, while the test set has a different distribution composed of molecules with more extreme HFE values.

For the third data split, we separate the data by chemical scaffold. It is designed to test the ability of the models to generalize to molecules with structures different from those seen during training. 320 molecules did not have a ring-based structure (were not classified within a molecular scaffold) and were randomly distributed between the train, test, and validation sets according the 6:1:1 ratio. The 322 ring-containing molecules (classified within a molecular scaffold) were split between the partitions, ensuring that all molecules within a given scaffold were in the same partition. Thus, each partition contains a distinct distribution of molecular scaffolds.

#### 2.4.2 Removal of Single Heavy Atom Molecules

Three molecules contained only one heavy atom and, as a result, their graph representations contain only one node. The MPNN is unable to handle graphs with a single node, and thus these molecules are excluded. For consistency, they were also excluded from training the GraphConv models. The molecules excluded are methane, hydrogen sulfide, and ammonia.

#### 2.4.3 Low Experimental Uncertainty Subset of Small Molecules

The experimental uncertainties of the HFE values listed in the database range from 0.03 to 1.93 kcal/mol, with a mean of 0.57 kcal/mol; for the majority (459 out of 642) of the molecules, the uncertainty is listed as a default value of 0.6 kcal/mol. Values with higher uncertainties are more likely to be inaccurate, meaning that if included in the training data, they could decrease the accuracy of DNN models. To decrease the likelihood of this, the molecules with the highest uncertainties were excluded. For molecules with |HFE| < 6 kcal/mol, the molecule was excluded if its uncertainty was greater than 0.6 kcal/mol. For molecules with |HFE| ≥ 6 kcal/mol, the molecule was excluded if its relative uncertainty was greater than 10%. This procedure removed 37 molecules and ensured that all remaining molecules have absolute uncertainty less than or equal to 0.6 kcal/mol or relative uncertainty less than or equal to than 10%.

After this filtering and the removal of molecules with only a single heavy atom, the remaining data set contains 602 molecules. The summary information for the data set before and after filtering can be seen in Table 3. This procedure modifies the sizes of the partitions for the above data splits, but the resulting partitions are close to the original 6:1:1 ratio. After filtering, the random stratified split has 450, 76, and 76 samples for the training, validation, and test sets, respectively. The split by HFE has 455, 79, and 68 molecules for train, validation, and test, while the scaffold split has 448, 78, and 76 molecules, respectively. The data is split before filtering out uncertainties so that accuracy of DNN models can be compared with and without including these high-uncertainty molecules. This analysis can be found in Section 3.1.

### 2.5 Training and Testing Protocol

For each combination of the data split and the physics-based model, DNN models were trained to correct for the error of the physics model. In each case, four different setups were tested as described in Section 2.3. For each setup, an ensemble of 20 models were trained individually. All 20 models are trained on the same training data, but each model uses a different random seed for weight initialization and stochastic gradient descent. The final prediction for each sample is calculated by averaging the predictions of each of the 20 models. It is well-known that even for highly expressive DNNs, using an ensemble of models to make predictions improves accuracy and generalization.^150^ All results presented in Section 3 use the average predictions from the 20 model ensembles. Due to the small data set, there is significant variation in accuracy between individual models. By using an ensemble, we average out these variations, resulting in a stronger and more robust final model.

The batch size was 100 and the learning rate was 0.001 for all models. Early stopping was used to end training when model performance converged on validation data to reduce the risk of significant overfitting. For the MPNN patience was set at 20 epochs and the minimum improvement necessary to continue training was set at 0.01 kcal/mol; if the validation set RMSE did not improve by at least 0.01 kcal/mol over a span of 20 epochs, training was stopped and the model was reverted back to the last epoch where the validation RMSE improved. The GraphConv model uses the same minimum improvement, but due to longer convergence time, the patience was set at 100 epochs.

## 3 Results and Discussion

Unless otherwise specified, all of the results presented in the following pertain to the relevant test set.

### 3.1 Improvement by Filtering out High-Uncertainty Samples

As seen in Figure 2, filtering out experimental values with high uncertainty results in lower RMSEs on the random stratified data split test set for both physics models and physics + DNN models. In addition, despite the lower physics model RMSEs, the relative improvement from DNN corrections is noticeably higher when only low uncertainty molecules are present. The degree of improvement varies between the physics models and DNN models, but the overall trend remains clear throughout. Across the 24 physics + DNN combinations, the relative improvement increases in almost all cases, and the mean relative improvement increases from 43.2% to 48.5%. Based on our analysis (results not shown), this improvement in accuracy comes from two sources. First, the removal of high-uncertainty data from the training prevents the model from becoming biased toward potentially unreliable experimental values. Second, the removal of these molecules from the test set prevents less reliable HFE values from increasing the RMSE. The effect of filtering out high-uncertainty data is inconclusive for out-of-distribution data, where the DNN models are inherently more inconsistent and less accurate. The complete results for all models trained with the full data set are shown in Tables S5–S7 in the Supporting Information. Given the positive effect of excluding high-uncertainty data points on in-distribution data, from here on we only use the low-uncertainty data set of 602 molecules, which means that roughly 6% of the original data set is excluded.

**Figure 2:**
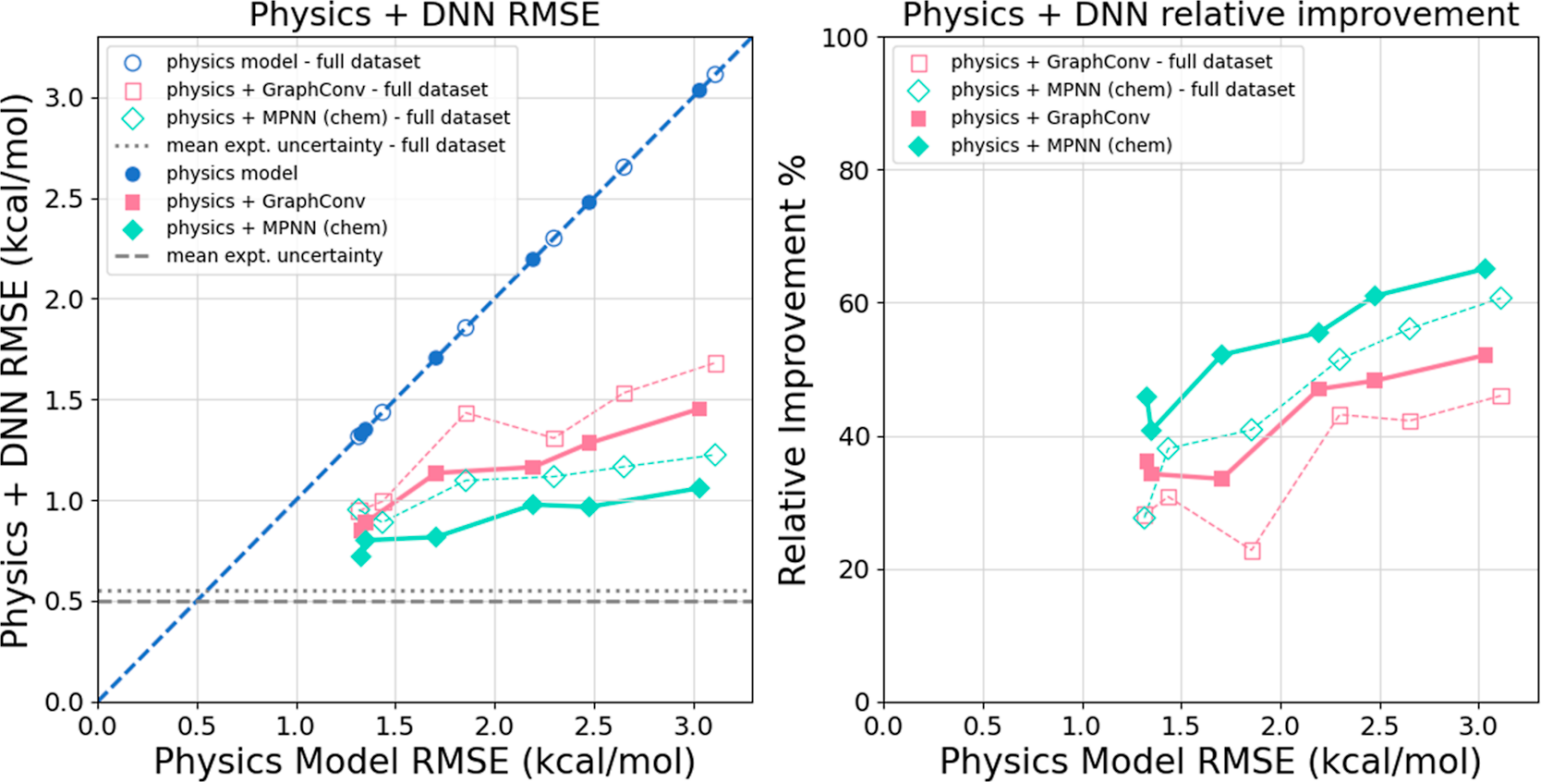
Physics + DNN models perform better when high uncertainty experimental values are excluded. (Left) Physics + DNN RMSE on the **random stratified data split** test set with and without filtering out high uncertainty experimental values plotted against physics model alone RMSE. The process for excluding uncertain experimental values is described in Section 2.4.3. Data points below the dashed blue line indicate that the physics + DNN model is more accurate than the physics model alone. In every case, the RMSE is lower when high uncertainty experimental values are excluded. (Right) Relative RMSE improvement for physics + DNN models on the **random stratified data split** test set with and without filtering out uncertain experimental values plotted against physics model alone RMSE. The relative improvement from DNN corrections is larger when high uncertainty experimental values are excluded.

### 3.2 GraphConv and MPNN Both Perform Well When Test Data Is Similar to Training Data

When using the random stratified data split, the RMSE to experiment for each physics + DNN model is significantly lower than the corresponding physics-based model alone, as seen in the left panel of Figure 3 and in Table 4. This result clearly shows that graph neural networks can significantly reduce error on unseen data. It is also clear that more accurate physics models result in more accurate physics + DNN models, highlighting the importance of using highly accurate physics models with this strategy. As seen in the right panel of Figure 3, the relative improvements were significantly lower for more accurate physics models. The two least accurate physics models, IGB5 and AASC, both had more than 60% relative improvement with the best DNN corrections, while the most accurate physics models still each had nearly 50% relative improvement for the best DNN models. After DNN corrections, the RMSE of all models is lower than or approaches 1 kcal/mol, with the most accurate models beginning to approach experimental uncertainty.

**Figure 3:**
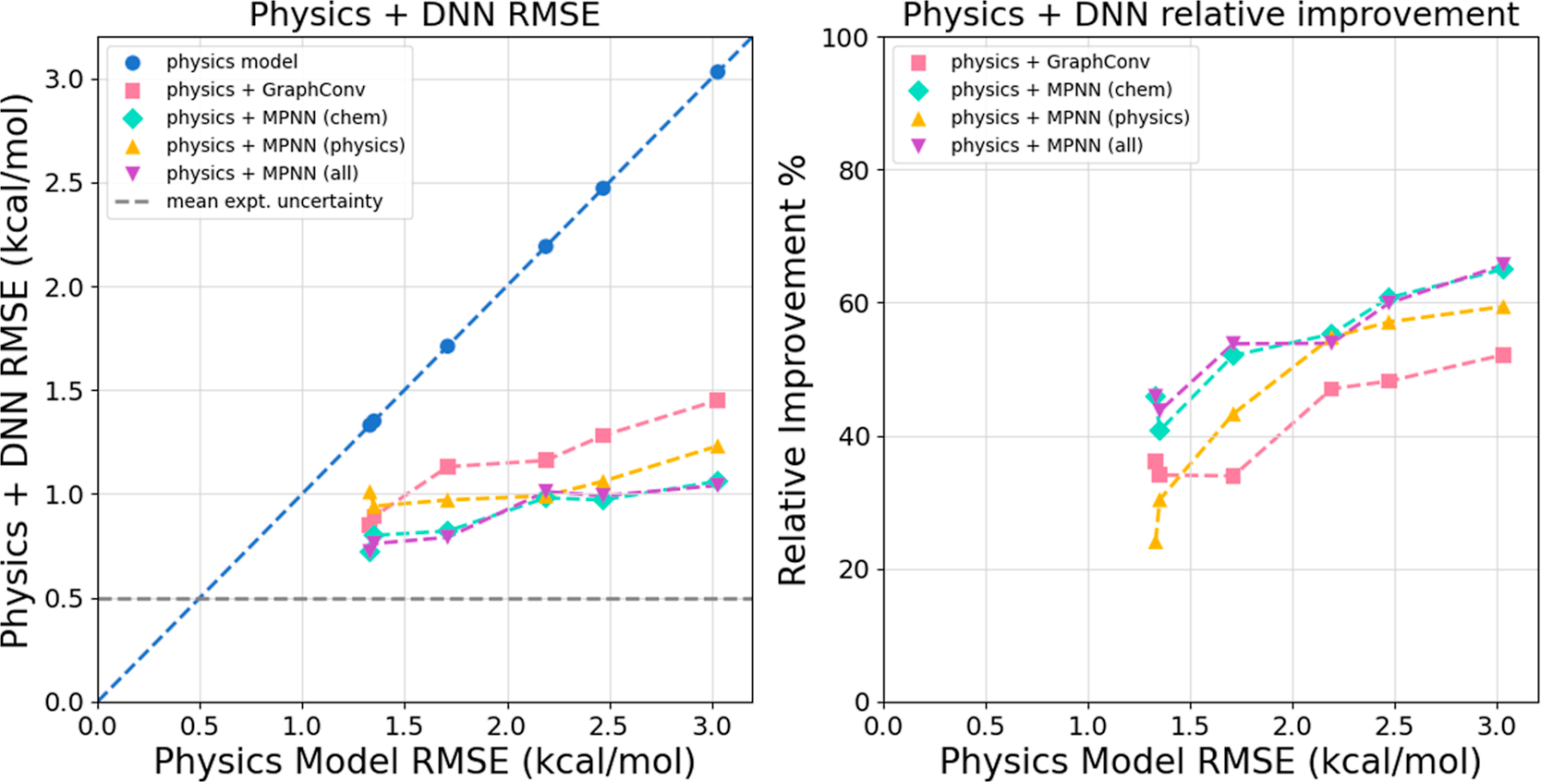
All DNN models significantly improve performance of physics models on the **random stratified data split** test set. (Left) Physics + DNN RMSE plotted against physics model alone RMSE. This figure visualizes the data presented in Table 4. Data points below the dashed blue line indicate that the physics + DNN model is more accurate than the physics model alone. Each physics + DNN model performs significantly better than its corresponding physics model alone. As the physics model accuracy improves, the accuracy of each corresponding physics + DNN model also improves and RMSE begins to approach experimental uncertainty (dashed gray line). (Right) Relative RMSE improvement for physics + DNN models plotted against physics model alone RMSE. The relative improvement from DNN corrections decreases but remains significant as the physics model accuracy increases.

**Table 4:** Performance of the Physics-Based Hydration Models with and without DNN Ensemble Corrections on the TEST Set of 76 Molecules Using the **Random Stratified Data Split** ^a^

physics model	TIP3P	AASC	CHA-GB	GBNSR6 (ZAP9)	GBNSR6 (mbondi)	IGB5	DNN alone
physics alone	1.35	2.47	1.33	1.70	2.19	3.03	N/A
physics + GraphConv	0.89	1.28	0.85	1.13	1.16	1.45	1.13
physics + MPNN (chem)	0.80	**0.97**	**0.72**	0.82	**0.98**	1.06	**1.01**
physics + MPNN (physics)	0.94	1.06	1.01	0.97	0.99	1.23	1.23
physics + MPNN (all)	**0.76**	0.99	**0.72**	**0.79**	1.01	**1.04**	1.07

a The details of the splitting procedure are found in Section 2.4.1. The performance metric is RMSE relative to the experimental HFEs in kcal/mol. In bold are the best performances for the correction of each physics model. The final column titled “DNN alone” shows the performance of each DNN when trained to predict HFE directly rather than predicting a correction to a physics model.

The best MPNN models offered a significant advantage over the GraphConv model, as seen in Figure 3 and Table 4. However, it is clear that both architectures are effective at significantly reducing physics model errors. When compared with GBRT results in Table S3, it is clear that the DNN models provide an appreciable accuracy advantage over GBRT.

The addition of physics-based features did not provide an improvement in performance. The MPNN (all) models with chemistry and physics features performed almost identically to the MPNN (chem) models with only chemistry-based features. This unexpected result can potentially be explained by the fact that the physics present in the physics-based features is already incorporated within the physics models. When only physics-based features were used, the performance was worse.

It is worth noting that the results using the GraphConv model here are somewhat better than was seen in Bass et al., which used an identical model architecture.^98^ For example, GBNSR6 (ZAP9) + DNN RMSE improves from 1.5 to 1.13 kcal/mol, while most other physics + DNN models improve slightly. These improvements can be explained by the use of ensembles and early stopping here in addition to the removal of high-uncertainty samples.

### 3.3 Performance of Physics + DNN on Data Different from the Training Data

#### 3.3.1 Error Reduction When Splitting the Data Set by HFE

On data with HFEs outside the range of training data, the best DNN models improve all physics model RMSEs, as seen in Table 5 and in the left panel of Figure 4. The improvements are substantial for less accurate physics models, while slight improvements were consistently seen even for the most accurate physics models. Nearly all physics + DNN model RMSEs are the range of 2 to 3 kcal/mol, a significant improvement over the 2.5 to 4.5 kcal/mol range seen by the physics models alone.

**Table 5:** Performance of the Physics-Based Hydration Models with and without DNN Ensemble Corrections on the TEST Set of 68 Molecules Using the Data **Split by HFE** Which Tests Performance on Out-Of-Distribution Data^a^

physics model	TIP3P	AASC	CHA-GB	GBNSR6 (ZAP9)	GBNSR6 (mbondi)	IGB5	DNN alone
physics alone	2.42	3.68	2.94	2.62	3.58	4.27	N/A
physics + GraphConv	**1.94**	2.39	**2.45**	**2.38**	2.31	**2.54**	6.48
physics + MPNN (chem)	2.01	**2.35**	2.66	2.66	**2.07**	2.64	**6.01**
physics + MPNN (physics)	2.26	2.37	2.81	2.84	3.11	2.88	6.67
physics + MPNN (all)	2.00	**2.35**	2.62	2.68	2.08	2.59	6.05

a The details of the splitting procedure are found in Section 2.4.1. The performance metric is RMSE relative to the experimental HFEs in kcal/mol. In bold are the best performances for the correction of each physics model. The final column titled DNN alone shows the performance of each DNN when trained to predict HFE directly rather than predicting a correction to a physics model.

**Figure 4:**
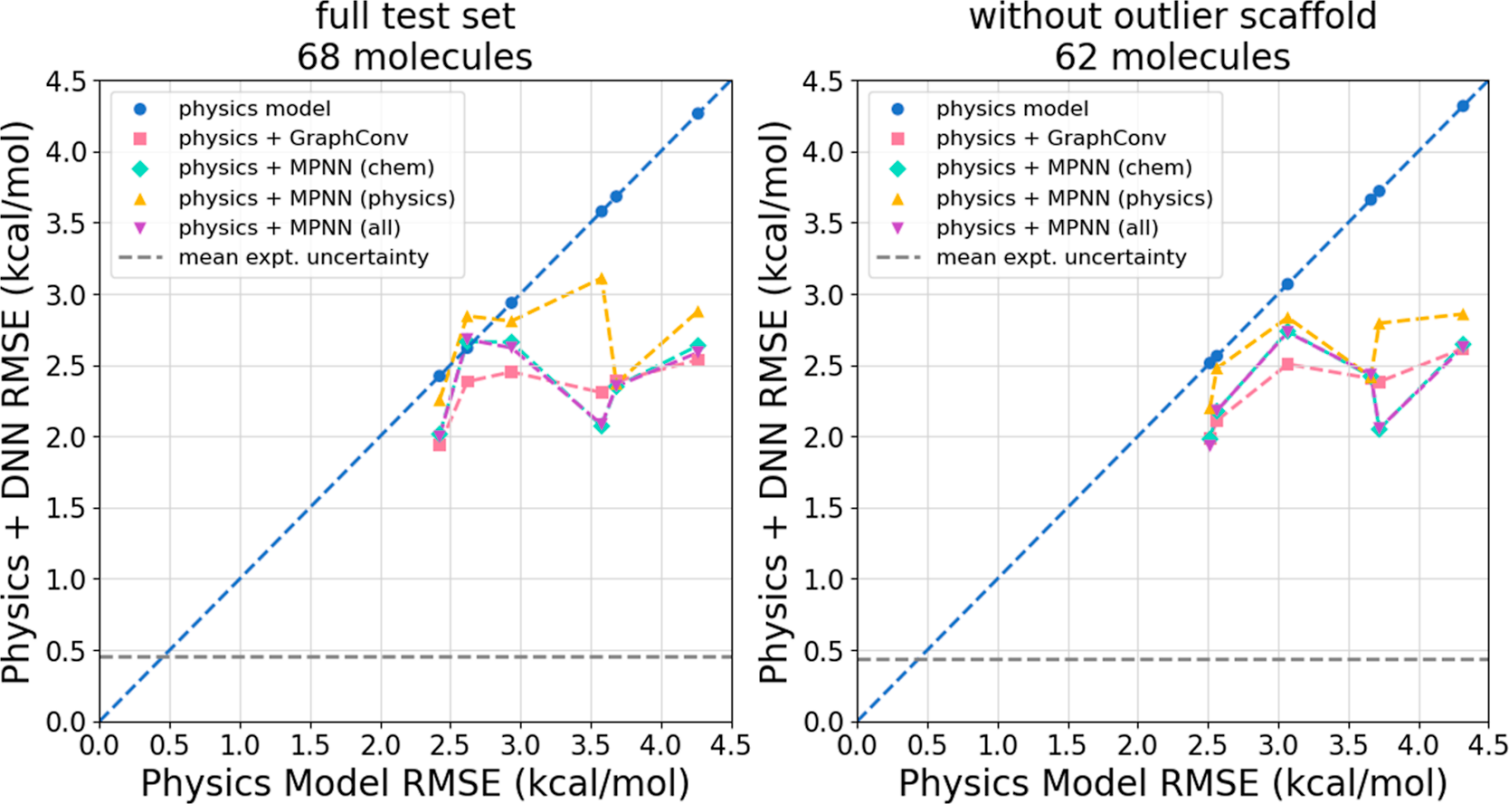
Use of DNN as a postprocessing correction improves the accuracy of the underlying physics model in almost all instances even for out of distribution HFEs. Shown are physics + DNN RMSE on the **split by HFE** test set plotted against physics model alone RMSE for each of the DNN models. (Left) Data for the entire test set of 68 molecules. This plot visualizes the data presented in Table 5. (Right) When excluding the 6 molecules in the O=c1cc­[nH]­c­(=O)­[nH]­1 scaffold, all DNN models improve the physics accuracy. Results for this outlier scaffold can be seen in the Supporting Information. Across both plots, data points below the dashed blue line indicate that the physics + DNN model is more accurate than the physics model alone. The least accurate physics models see significant improvement due to DNN corrections while the more accurate physics models see much smaller improvements.

More accurate physics model predictions tend to lead to more accurate overall predictions; the most accurate model (TIP3P) corresponds with the best physics + DNN result while the least accurate model (IGB5) corresponds to the worst physics + DNN result. However, this ranking trend is not as strong as for in-distribution data as seen above. We believe that this weaker trend can be explained by data set limitations. First, the data set is small, which means that the real trends may be partially hidden by statistical noise. Additionally, the differing distributions of data between train and test sets may limit final accuracy making it difficult to achieve performance better than a given threshold (around 2 kcal/mol RMSE here). These limitations with the data set make it hard to draw any stronger conclusions on the impact of the accuracy of the physics model on predictions for molecules with out-of-distribution HFEs.

Similar to what has been seen with the random data split, the physics features seem to provide no real added value when combined with chemistry features, while model performance is poor when only using physics features. Somewhat surprisingly, the MPNN and GraphConv models perform very similarly here with no notable advantage for one over the other. The GBRT models also perform similarly to the DNN models as seen in Table S3.

A closer look at Table 5 and the left panel of Figure 4 shows that in a few cases DNN slightly worsens accuracy and in some other cases offers almost no improvement. This result can be largely explained by the poor accuracy of the physics + DNN models on the 6 molecules in the O=c1cc­[nH]­c­(=O)­[nH]­1 scaffold. When excluding these 6 molecules, *every* DNN model improves the physics accuracy, and the improvement is at least 0.3 kcal/mol in almost all cases.

Figure 5 shows how the different GBNSR6 (mbondi) + DNN models perform across the entire range of molecules in the test set. The DNN models perform well on data near the test set as many particularly poor physics predictions near the train–test cutoff are significantly reduced. However, for molecules with HFE significantly further from the training set distribution, the effect becomes less clear. In many cases, the DNN corrections improve the prediction while worsening it in other cases. The overall trends are similar for other physics models, as seen in Figure S5 in the Supporting Information.

**Figure 5:**
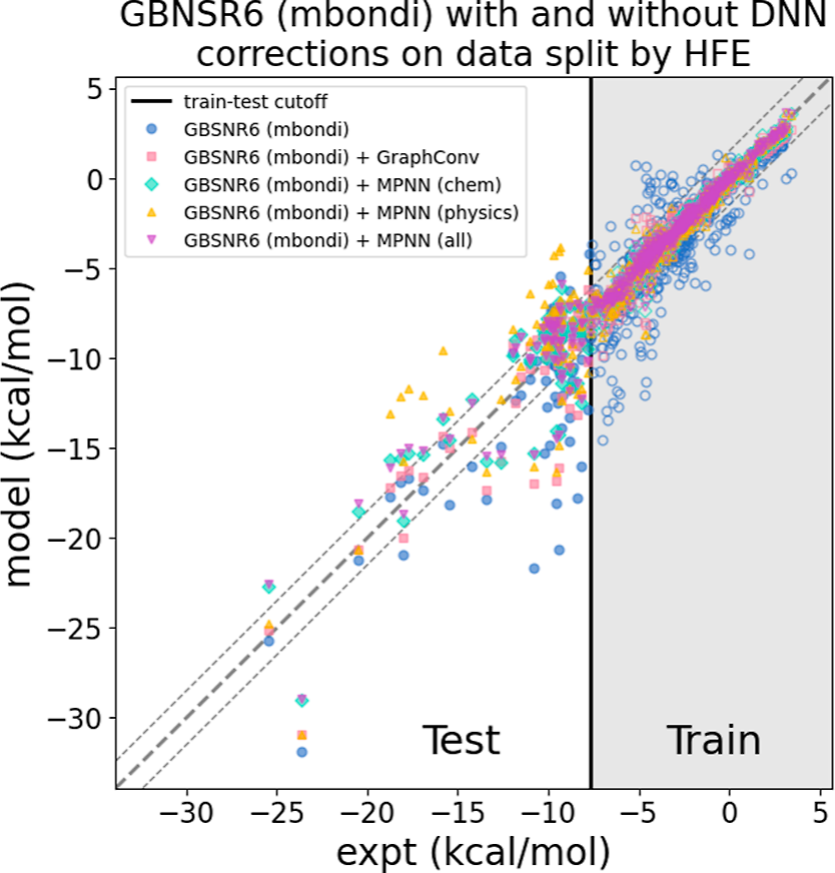
DNN corrections improve physics-based predictions (GBNSR6, mbondi) on molecules with out-of-distribution HFE; the accuracy benefit of the correction diminishes for the most extreme HFE values. Data to the right of the vertical line (hollow data points) are those included in the training set while data to the left of the line (solid) are used as the test set. The thick dashed line indicates experiment while the thinner dashed lines show experiment ±1.5 kcal/mol. In most cases, physics predictions outside these lines (blue points) are “pushed” closer to the experimental reference by the DNN corrections.

The benefits of the DNN corrections will likely be small, if any, for molecules very far away from the training set. However, from these results, we can conclude that the physics + DNN results will likely perform, on average, at least as good as and in many cases significantly better than the physics model on molecules with out-of-distribution HFE.

##### 3.3.1.1 Comparison with DNN Alone Results

Our previous work showed that DNN alone struggled to generalize to blocked amino acids, which generally have higher absolute HFEs than training data.^98^ Here we test DNN alone on data partitioned by HFE to confirm the general finding of poor accuracy of DNN alone on samples with out-of-distribution HFE as seen in Figure 6. The models trained using Set2Set node aggregation, the scheme which was used throughout this work, fail to display any ability to predict out-of-distribution HFEs as seen in the left panel of Figure 6. Based on this, we hypothesize that the Set2Set aggregation may not be optimal for the DNN alone when making predictions for out-of-distribution HFEs. To test whether this poor performance is due to the Set2Set procedure, we tested an alternative aggregation scheme by summing all node features in a graph. When using sum aggregation, DNN models are able to generalize to some extent but struggle greatly to accurately predict HFE, particularly far outside the training set distribution. The full results showing the RMSE of all models using sum aggregation are shown in Table S4 in the Supporting Information. Even in this case, all physics + DNN models significantly outperform DNN alone. From this result, it is clear that the Physics + DNN strategy is far superior to DNN alone for samples outside the training set distribution.

**Figure 6:**
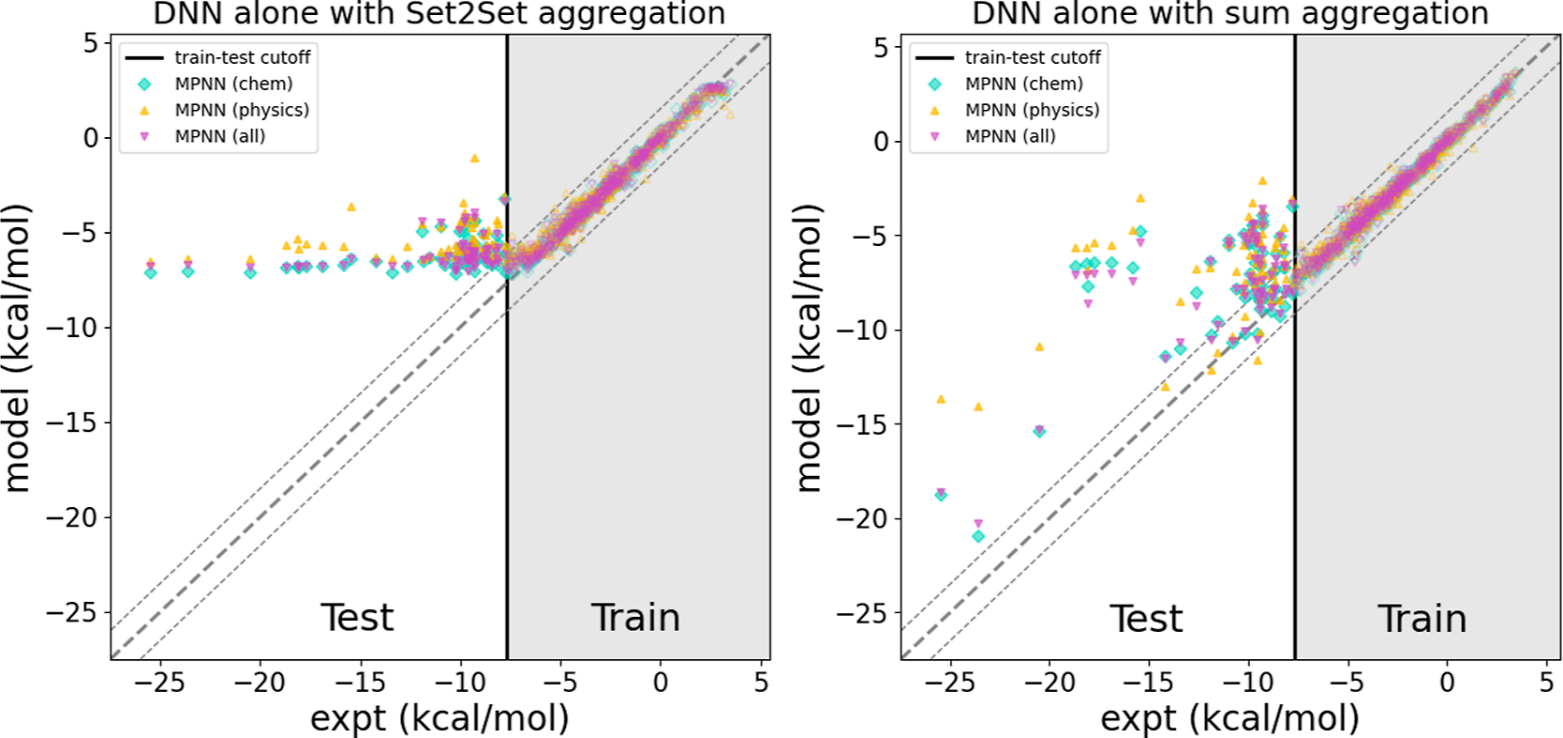
DNN alone models struggle to perform well on molecules with out-of-distribution HFE. When this work’s default Set2Set node aggregation scheme is used (Left), the models are unable to generalize at all. When we use sum node aggregation (Right) the DNN predictions are more accurate, but still generalize poorly overall. Data to the right of the vertical line (hollow data points) are those included in the training set while data to the left of the line (solid) are used as the test set. The thick dashed line indicates experiment while the thinner dashed lines show experiment ±1.5 kcal/mol. Even for the sum node aggregation scheme, which is best suited for DNN alone approach (right panel) most DNN predictions are outside of these lines.

#### 3.3.2 Error Reduction when Splitting the Data Set by Molecular Scaffold

The physics + DNN models perform significantly better than their physics model counterparts on the scaffold data split test set, as seen in Table 6 and Figure 7. In many cases, the RMSE is actually lower for this test set than for the random stratified test set seen in Table 4. However, this can be primarily attributed to the lower physics model RMSE on these data. Additionally, the test set is composed of half of the molecules without ring-based structures (not in a molecular scaffold) while only half of the molecules are in a scaffold differing from the train set. When looking at just molecules in unseen molecular scaffolds, seen in the right panel of Figure 7, it becomes clear that the accuracy here is worse than when using the random data split in Section 3.2.

**Table 6:** Performance of the Physics-Based Hydration Models with and without DNN Ensemble Corrections on the TEST Set of 76 Molecules Using the **Scaffold Split** Which Tests Performance on Out-Of-Distribution Data^a^

physics model	TIP3P	AASC	CHA-GB	GBNSR6 (ZAP9)	GBNSR6 (mbondi)	IGB5	DNN alone
physics alone	1.40	2.06	1.76	1.44	1.78	2.34	N/A
physics + GraphConv	0.90	0.98	**1.30**	1.06	0.93	**0.89**	1.52
physics + MPNN (chem)	1.09	0.82	1.39	0.96	0.91	1.00	1.32
physics + MPNN (physics)	**0.89**	1.27	1.32	**0.76**	**0.79**	1.21	1.40
physics + MPNN (all)	1.12	**0.79**	1.33	0.96	0.89	0.92	**1.12**

a The details of the splitting procedure are found in Section 2.4.1. The performance metric is RMSE relative to the experimental HFEs in kcal/mol. In bold are the best performances for the correction of each physics model. The final column titled DNN alone shows the performance of each DNN when trained to predict HFE directly rather than predicting a correction to a physics model.

**Figure 7:**
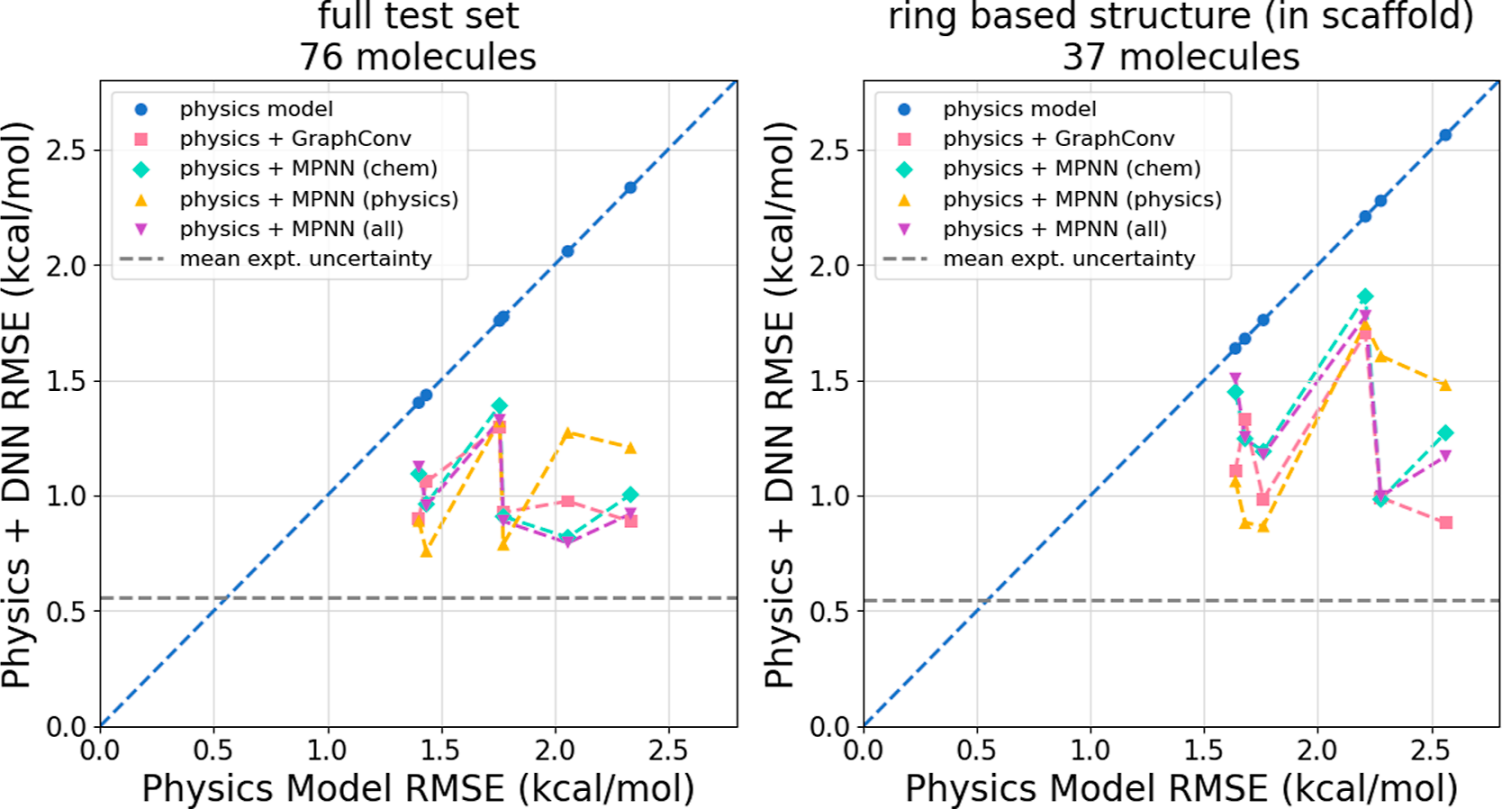
Physics + DNN RMSE on the **scaffold split** test set plotted against physics model alone RMSE for each of the DNN models. (Left) Data for the entire 76 molecule test set including molecules with no rings. This plot visualizes the data presented in Table 6. (Right) Data for the 37 molecules with ring based structures that are in a molecular scaffold. For both plots, data points below the dashed blue line indicate that the physics + DNN model is more accurate than the physics model alone. Even when predicting HFEs for molecules with unseen scaffolds, each physics + DNN model performs significantly better than its corresponding physics model alone.

The results are also inconsistent between different physics models, with no clear trend showing that more accurate physics models lead to better final physics + DNN predictions. This lack of a clear trend is likely due to noise as a result of the small data set, making it is difficult to make stronger conclusions on the impact of physics model accuracy on predictions for molecules with out-of-distribution molecular scaffolds. The use of physics-based features provided minimal improvement when combined with chemistry features. As seen in Table 6, when the physics features were used alone, the results were very inconsistent, performing best for some physics models and worst for others. The GraphConv and MPNN models perform similarly here with no clear advantage for either, but both significantly outperform the GBRT models.

Figure 8 shows how the GBNSR6 (mbondi) + DNN models perform across the entire scaffold split train and test sets. The models consistently improve inaccurate predictions on unseen scaffolds in the test set. We can conclude that the DNN corrections offer significant improvements to the physics predictions on molecules with scaffolds different from the training data. This makes sense since the molecules have HFEs and composition similar to those of the training data. The overall trends are similar for other physics models, as seen in Figure S6 in the Supporting Information.

**Figure 8:**
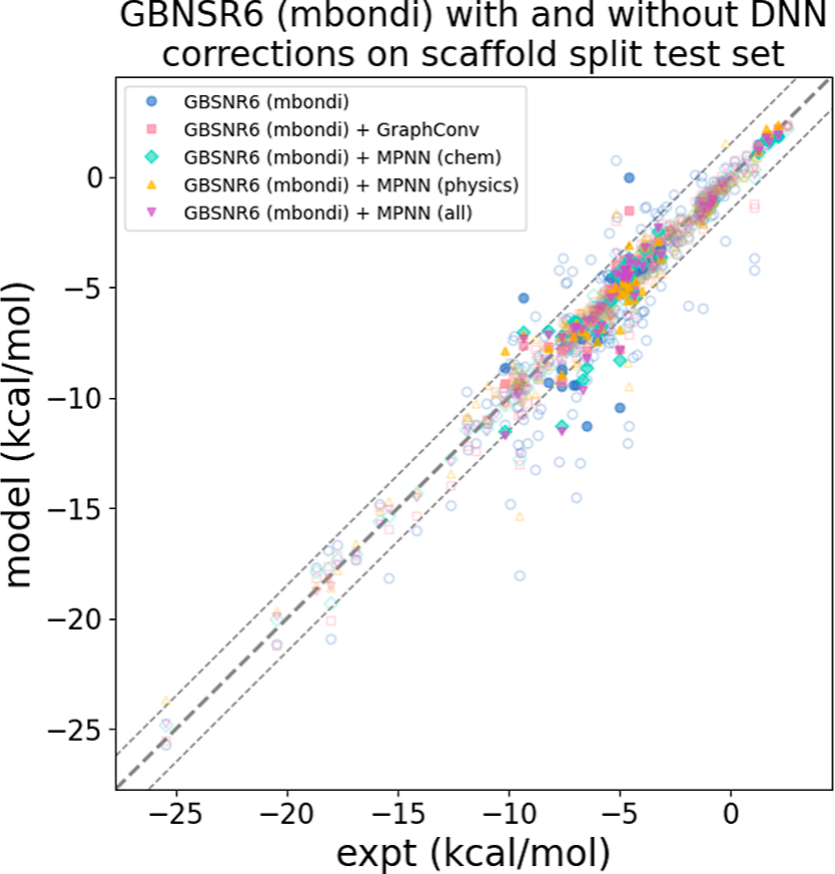
DNN corrections improve predictions by physics-based model (GBNSR6, mbondi) on molecules with unseen molecular scaffolds. Hollow data points are the 215 molecules with molecular scaffolds included in the training set while the 37 molecules with unseen molecular scaffolds are shown as solid points. Note that molecules without a ring based structure (not in a scaffold) are not included in this figure. The thick dashed line indicates experiment while the thinner dashed lines show experiment ±1.5 kcal/mol. In most cases, physics predictions outside these lines (blue points) are pushed closer to experiment by the DNN corrections.

## 4 Conclusions

Accurate prediction of hydration free energy remains a critical challenge in molecular modeling, impacting diverse applications such as drug discovery and biomolecular simulations. Computational models based on physics are generally not specific to a particular set of molecules and are therefore expected to deliver results that make sense for a wide range of biomolecules. However, depending on the level of their physical realism, these models can become prohibitively expensive computationally, especially in the context of high-throughput studies. Some physics-based models are computationally efficient, but these models often lack the accuracy required for nuanced predictions. On the other hand, purely data-driven machine learning (ML) approaches, although capable of capturing subtle patterns in large data sets, struggle with generalization beyond the training set distribution. This work addresses this gap by proposing a hybrid approach that leverages the strengths of both methodologies. Specifically, we demonstrate the utility of employing deep graph neural networks as an independent postprocessing correction step to improve the hydration free energy (HFE) predictions of physics-based water models.

Our results highlight several key findings. Most importantly, for molecules outside the training set distribution, the hybrid approach provides significant improvements over the physics-only models, albeit with reduced consistency for extreme outliers. Even far outside the distribution of the training set, DNN corrections, on average, improve the accuracy of physics-based predictions. This result is consistent across two different data set splits designed to test generalization to out-of-distribution samples. Importantly, our approach also consistently outperforms DNN-alone models, which struggle to generalize to out-of-distribution data. For molecules within the training set distribution, DNN corrections reduced the RMSE to below 1 kcal/mol for most physics-based models, approaching experimental uncertainty in many cases. These results emphasize the potential of this specific strategy of combining physics-based priors with data-driven flexibility, yielding a robust strategy that maintains the reliability of physical constraints while enhancing overall accuracy.

The main significance of this work lies in the reliability of using DNN as an independent postprocessing correction step for reasonably accurate physics-based predictions even when the size of experimental data sets are very limited, which is expected to be the case in this, and many related fields for the foreseeable future. This strategy is inherently unlikely to produce nonsensical predictions, even for data unseen during training, provided that the underlying physics model provides a reliable baseline and the experimental values are trustworthy. Due to the relatively high accuracy of the physics models used in this work, the DNN corrections remain small relative to the physics contribution, which ensures that the overall prediction preserves the physical trends outside the training set distribution. The DNN alone models struggle to generalize to out-of-distribution samples and, in many cases, provide highly inaccurate predictions. In contrast, our physics + DNN approach avoids highly unrealistic predictions while improving accuracy on average even for out-of-distribution data. Of note, our key result that the physics + DNN as a postprocessing step improves physics-based predictions on out-of-distribution data remains valid if DNN is replaced with gradient boosted regression model, with the caveat that the overall accuracy of the DNN-based approach is higher.

We have attempted to enhance the reliability of the experimental HFE values by removing a small percentage of values with the highest experimental uncertainty, which improves the predictive accuracy of the models for in-distribution data, while the effect on out-of-distribution accuracy is inconclusive. For the in-distribution data, the removal of high-uncertainty data from the training set and the test set both contribute to the accuracy improvement. This observation supports the idea that the quality and reliability of ground-truth labels may be just as important as simply increasing the overall data set size. However, the inherent limitations of our data set do not allow us to draw strong quantitative conclusions here; we suggest that further study is required to fully understand the impact of accuracy of ground truth labels on the training and performance of the DNN model.

The proposed approach has several limitations. The accuracy gains diminish for molecules with HFEs far outside the training distribution, where the DNN corrections can sometimes introduce new errors. This result underscores the need for caution when applying this method to individual molecules, as some predictions can worsen even though overall performance improves. Additionally, while physics + DNN models performed well on molecular scaffolds, the magnitude of improvement was lower compared to in-distribution data, and results were less consistent across different physics models. Finally, we did not test the approach for very low-quality (but potentially very fast) physics-based models, where the DNN correction is expected to carry more weight. The mere fact that the starting model is physics-based may not be enough to guarantee the safety of the proposed combination approach in the sense discussed above.

Future directions for addressing these limitations include exploring data augmentation techniques, such as incorporating multiple molecular conformations to enhance model robustness, and adopting advanced ML architectures, such as equivariant graph neural networks or graph transformers. Additionally, future work can include applying this strategy to other problems, such as using DNNs to improve physics-based protein–ligand binding affinity predictions.

In summary, the physics + DNN as a postprocessing step framework offers a practical, scalable, and effective solution to efficiently improve the accuracy of hydration free energy predictions. By demonstrating its success across multiple data distributions, including unseen molecular scaffolds and out-of-distribution HFEs, this work shows the potential of this strategy for biomolecular modeling and simulations where solvent effects are of critical importance, such as in improving the accuracy and efficiency of ligand binding predictions. Broadly speaking, this work suggests a promising approach to combine physics and DNN in a “safe” way, potentially useful in other areas.

## Supplementary Material



## Notes

Details of the physics-based models of solvation; additional tables and figures; access to open source code (PDF)
