# Supplementary material for: Using Deep Graph Neural Networks Improves Physics-Based Hydration Free Energy Predictions Even for Molecules Outside of the Training Set Distribution
Source: J Phys Chem B. 2025 Jul 11;129(29):7483–98. doi: 10.1021/acs.jpcb.5c02263 (PMC12302205; doi:10.1021/acs.jpcb.5c02263)
Supplement: Supplementary file 1 [file jp5c02263_si_001.pdf]

Supporting Information for "Using Deep Graph Neural Networks Improves Physics-Based Hydration Free Energy Predictions Even for Molecules Outside of the Training Set Distribution"

Luke H. Elder[†] and Alexey V. Onufriev[*,†,‡,¶]

†Department of Computer Science, Virginia Tech, Blacksburg, VA 24061, United States

‡Department of Physics, Virginia Tech, Blacksburg, VA 24061, United States

¶Center for Soft Matter and Biological Physics, Virginia Tech, Blacksburg, VA 24061, United States

E-mail: alexey@cs.vt.edu

1 The Physics-based Water Models and their Parameters. Details.

1.1 TIP3P

TIP3P[1] is a classical, fixed-charge (non-polarizable) rigid, 3-point, explicit water model. The model geometry closely approximates experimental values[2] for water molecule in gas phase. Despite its respectable age, the model is still widely used in biomolecular simulations for a number of reasons,[3] including good performance in estimates of HFEs. The availability of

pre-computed HFEs for all of the small molecules in FreeSolv[4] makes the choice particularly attractive, given the significant computational expense associated with these calculations.[5]

1.2 IGB5 (GB-OBC)

The IGB5 (GB-OBC)[6] generalized Born (GB) model from AMBER[7] originally parameterized against reference numerical Poisson-Boltzmann (PB) hydration free energies calculated under the standard assumption that the dielectric boundary (DB) is the solvent excluded surface (SES) determined by Bondi radii and water probe radius $r_w = 1.4$ Å.

In GB-OBC the polar component of the solvation energy, ΔG_{pol}, is approximated by the canonical GB equation:[8]

$$\Delta G_{pol} = -\frac{1}{2}\left(\frac{1}{\epsilon_{\text{in}}} - \frac{1}{\epsilon_{\text{out}}}\right)\sum_{ij}\left(\frac{q_i q_j}{f_{ij}^{\text{GB}}}\right), \tag{1}$$

where $\epsilon_{\text{in}} = 1$ and $\epsilon_{\text{out}} = 80$ are the dielectric constants of the solute and the solvent, respectively, q_i are the charges on the solute atoms. The method employs the most widely used functional form of f_{ij}^{GB}:

$$f_{ij}^{\text{GB}} = \left[r_{ij}^2 + R_i R_j \exp(-r_{ij}^2/4R_i R_j)\right]^{\frac{1}{2}}, \tag{2}$$

where r_{ij} is the distance between atomic charges q_i and q_j, and R_i, R_j are the so-called *effective Born radii* of atoms i and j, which represent each atom's degree of burial within the solute. They are calculated using an analytical approximation for the r^{-4} integral over the molecular volume.

1.3 GBNSR6

The Generalized Born Numerical Surface R6 integration (GBNSR6) method[9,10] addresses the systematic error (with respect to PB results)[11] and the known peptide secondary structure

bias[12] of the classical GB models based on Still's original[8] equation 1 (IGB5 (GB-OBC) models[6]).

An efficient computation of total solvation free energies is performed with an MSMS-based implementation of GBNSR6.[10] The polar component of the solvation energy, ΔG_{pol}, is calculated by the modification[13,14] of the generalized Born[8] model:

$$\Delta G_{pol} = \sum_{ij} \Delta G_{ij}^{pol} \approx -\frac{1}{2}\left(\frac{1}{\epsilon_{\text{in}}} - \frac{1}{\epsilon_{\text{out}}}\right) \frac{1}{1+\beta\alpha} \sum_{ij} q_i q_j \left(\frac{1}{f_{ij}^{\text{GB}}} + \frac{\alpha\beta}{A}\right), \quad (3)$$

where $\beta = \epsilon_{\text{in}}/\epsilon_{\text{out}}$, $\alpha = 0.571412$, and A is the electrostatic size of the molecule, which is essentially the overall size of the structure that can be computed analytically. f_{ij}^{GB} function is defined as in the IGB5 (GB-OBC) model, by Eq. 2, where the effective Born radii R_i are calculated by the "$R6$" equation:[9,10,15,16]

$$R_i^{-3} = \left(-\frac{1}{4\pi} \oint_{\partial V} \frac{\mathbf{r} - \mathbf{r_i}}{|\mathbf{r} - \mathbf{r_i}|^6} \cdot d\mathbf{S}\right), \quad (4)$$

where ∂V represents the chosen representation of the dielectric boundary (DB) of the molecule, $\mathbf{r}$ represents the position of the infinitesimal surface element, $d\mathbf{S}$ is the infinitesimal surface element vector at $\mathbf{r}$, and $\mathbf{r_i}$ is the position of atom i. To determine the dielectric boundary ZAP9 atomic radii were used which produces a more accurate values of ΔG_{solv} with respect to experimental data compared to other commonly used radii.[10] Standard water probe radius $r_w = 1.4$ Å is used as well. The MSMS package[17] was employed to build a numerical triangulation of the Lee-Richards[18] molecular surface. The empirical constant offset B to the inverse radii[16] is set to 0, based on the recommendation in.[10] A grid-based surface GBNSR6[19,20] is available in AmberTools 2015[21] and later versions.

The nonpolar component of the solvation free energy, ΔG_{nonpol}, is computed using the approach proposed in Ref.:[22]

$$\Delta G_{nonpol} = \Delta G_{cav} + \Delta G_{\text{vdW}}, \quad (5)$$

where ΔG_{nonpol} is decomposed into cavity ΔG_{cav} and van der Waals (vdW) ΔG_{vdW} terms. The cavity component is calculated by:

$$\Delta G_{\text{cav}} = \gamma \cdot SASA, \tag{6}$$

where γ is the surface tension parameter and $SASA$ is the solvent accessible surface area. An optimal value $\gamma = 0.01$ kcal/mol/Å^2 is used based on the calculations described in Ref.[10] The solute-solvent van der Waals interaction term is calculated as:[22,23]

$$\Delta G_{\text{vdW}} = \sum_i \mu_i \frac{a_i}{(R_i + \rho_w)^3}, \tag{7}$$

where μ_i is a dimensionless adjustable parameter that depends on the atom type, ρ_w is the water probe radius, R_i is the effective Born radius of atom i. The values of a_i in Eq. 7 are computed as:[22]

$$a_i = -\frac{16}{3}\pi d_w \epsilon_{iw} \sigma_{iw}^6, \tag{8}$$

where $d_w = 0.033428$ Å^{-3} is the number density of water at standard conditions, ϵ_{iw} and σ_{iw} are calculated as:

$$\sigma_{iw} = \frac{1}{2}(\sigma_i + \sigma_w), \tag{9}$$

$$\epsilon_{iw} = \sqrt{\epsilon_i \epsilon_w}, \tag{10}$$

where $\sigma_w = 3.1507$ Å and $\epsilon_w = 0.1520$ kcal/mol are the Lennard-Jones parameters of the TIP3P water oxygen atom. σ_i and ϵ_i are the Lennard-Jones parameters for solute atom i. The values of σ_i, ϵ_i and μ_i for each atom type are presented in Table S1.

1.4 CHA-GB

The Charge Hydration Asymmetric Generalized Born (CHA-GB) method[23] introduces charge hydration asymmetry upon solute charge inversion, missed in the standard linear response

Table S1: Lennard-Jones parameters used for the calculation of $\Delta G_{\rm vdW}$ with the ZAP-9 radii set. A comment on some of the optimized μ_i values being negative is made in Ref.[10]

	σ_i (Å)	ϵ_i (kcal/mol)	μ_i
H	2.64953	0.0157	-0.0484908
C	3.39967	0.1094	0.129509
N	3.2500	0.1700	0.186843
O	2.9592	0.2100	0.1511
S	3.56359	0.2500	0.0136324
P	3.74177	0.2000	-0.051784
F	3.11815	0.0610	0.0416579
Cl	3.47094	0.2650	0.0840834
Br	3.95559	0.3200	0.00958446
I	4.18722	0.4000	-0.0264674

continuum electrostatics theory used in the PE or standard GB methods. The CHA effects are observed experimentally and when using most of the explicit water models. The asymmetry originates from the interaction of solute charges with the cubic octupole moments of water molecules surrounding the solute.

In CHA-GB approach, the dielectric boundary (DB) around solute, used to calculate the effective Born radii R_i (Eq. 4), is shifted from the solute atoms by increasing their atomic radii by $R_s = 0.52$ Å. This shifted boundary represents so-called "charge-symmetric" DB, responsible for the "charge-symmetric" part of the dielectric response of the solvent. Then, the "charge-symmetric" R_i are used to calculate "charge-asymmetric" effective Born radii $\tilde{R}_i$ by scaling each R_i as:

$$\tilde{R}_i = R_i \left(1 + Sgn \left[\sum_j q_j e^{-\tau \frac{r_{ij}^2}{R_i R_j}} \right] \frac{R_{OH}^z}{R_i - R_s + \rho_w} \right). \tag{11}$$

Here, parameter $R_{OH}^z = 0.5859$ Å characterizes asymmetry of the charge distribution in a water molecule and is a ratio of its primitive quadrupole and dipole moments, $\tau = 1.47$ is the parameter that controls an effective range of the influence of neighboring (to i) solute charges (j) on the sign of the CHA-correction in the R_i scaling factor.

The polar component of the solvation free energy, ΔG_{pol}, is calculated using Eq. 3, where

the empirical function f_{ij}^{GB} is replaced by $f_{ij}^{\mathrm{CHA-GB}} = \left[r_{ij}^2 + \tilde{R}_i\tilde{R}_j \exp(-r_{ij}^2/4R_iR_j)\right]^{\frac{1}{2}}$.

The nonpolar component of the solvation energy, ΔG_{nonpol}, is computed as in GBNSR6 method above, using Eqs. 5, 6 and 7, but with $\gamma = 0.0178$ kcal/mol/Å^2 and different values of μ_i (see Table 6 in the SI for Ref.[23]).

1.5 Analytical Apparent Surface Charge (AASC)

The AASC[24] method provides an approximate, boundary element formulation for the apparent surface charge at the dielectric boundary (DB) of a molecule. Taking advantage of a differentiable expression for the electrostatic potential around a molecule,[25,26] the corresponding closed-form expression for the apparent surface charge (ASC) at a point $\mathbf{r}$ at the DB is:

$$\sigma(\mathbf{r}) = \left(\frac{1}{\epsilon_{\mathrm{in}}} - \frac{1}{\epsilon_{\mathrm{out}}}\right)\left(\frac{1}{4\pi\,(1+\alpha\beta)}\right)\sum_i q_i \left[\left(\frac{\alpha\,(1-\beta)}{A^2}\right) - \cos(\varphi_i(\mathbf{r}))\left(\frac{1+\alpha}{d_i^2(\mathbf{r})}\right)\right], \quad (12)$$

where $\epsilon_{\mathrm{in}}, \epsilon_{\mathrm{out}}, \beta$, and A are as in GBNSR6 Sec. 1.3, $\alpha = 0.580127$, $\varphi_i(\mathbf{r})$ is the angle between the outward facing surface normal at the surface patch at $\mathbf{r}$ and the distance vector $\mathbf{d}_i(\mathbf{r})$ from the solute point charge q_i at $\mathbf{r}_i$ to the surface patch at $\mathbf{r}$. Once available, the ASC is used to compute the electrostatic solvation free energy of a molecule as:

$$\Delta G_{\mathrm{el}} = \frac{1}{2}\sum_i q_i \oint_{\partial V} \frac{\sigma(\mathbf{r})}{|\mathbf{r}_i - \mathbf{r}|}\, d^2s. \quad (13)$$

In this work, corresponding AASC nonpolar solvation free energies are taken to be the cavity component (Eq. 6) of Eq. 5 with, once again, $\gamma = 0.01$ kcal/mol/Å^2.

Numerically, the surface integral in Eq. 13 is computed using a triangulated surface mesh. We compute the solvent excluded surface (SES) mesh via NanoShaper[27] using a 1.4 Å water probe and Bondi[28] radii. Employing the SES as the dielectric boundary (DB), we can

approximate Eq. 13 as:

$$\Delta G_{\text{el}} \approx \frac{1}{2}\sum_i \sum_T \frac{q_i \sigma_T A_T}{|\mathbf{r}_i - \mathbf{r}_T|}, \tag{14}$$

where $\mathbf{r}_T$, A_T and σ_T are the position of the center, area and ASC of the triangle T on the DB, respectively. Augmenting the Bondi atomic radii with our choice of water probe above, the SAS can be computed as the numerical probe is taken to 0; in this limit, the interface surface area computed by NanoShaper is the SASA. The NanoShaper grid scale employed in AASC solvation energy calculations is 2, corresponding to a maximum triangular side length of $\frac{\sqrt{3}}{2}$ Å in the generated mesh.

2 Gradient Boosted Regression

2.1 Methods

To compare with our physics + DNN results, we trained gradient boosted regression trees (GBRT)[29,30] to correct for the physics model errors. We use Extended-Connectivity Fingerprints (ECFP)[31] and specifically ECFP4 fingerprints generated by RDKit to featurize the molecules. We use the GradientBoostedRegressor implemented within scikit-learn.[32] A preliminary exploration of hyperparameters indicated that model performance was largely unaffected by the specific values selected. We use primarily default hyperparameters, which are shown in Table S2, to ensure a fair comparison with our GNN models for which we did not optimize hyperparameters. We use the same three test sets as described in the main text, and use the same validation data for early stopping to determine the number of estimators used. The patience for early stopping was extended to 40 iterations to better ensure convergence of each model and the tolerance for early stopping is set to 0.01 kcal/mol. As with the DNNs, we train 20 independent models which are used to create an ensemble for final predictions.

Table S2: Hyperparameters used for the GBRT models implemented in scikit-learn.

hyperparameter	value
loss	squared_error
learning_rate	0.1
subsample	1.0
criterion	friedman_mse
min_samples_split	2
min_samples_leaf	1
min_weight_fraction_leaf	0.0
max_depth	3
min_impurity_decrease	0.0

2.2 Results

As seen in Table S3, accuracy of physics + GBRT models is generally good. However, in most cases, accuracy is noticeably worse than the physics + MPNN (chem) models which were chosen as a respresentative DNN model for comparison. For the random stratified split and the scaffold split, the GBRT models perform worse in all cases. For the split by HFE, the gap in the accuracy is smaller and in three cases, the physics + GBRT model is slightly more accurate.

Table S3: In nearly every case, physics + MPNN (chem) outperforms physics + GBRT on all three test sets. The split by HFE and molecular scaffold split test performance on out-of-distribution data. The details of the splitting procedure are found in the main text. The performance metric is RMSE relative to the experimental HFEs in kcal/mol. In bold are the best performances for the correction of each physics model and data split combination. The final column titled “ML Alone" shows the performance of each DNN or GBRT when trained to predict HFE directly rather than predicting a correction to a physics model.

split	model	TIP3P	AASC	CHA-GB	GBNSR6 (ZAP9)	GBNSR6 (mbondi)	IGB5	ML Alone
random	physics	1.35	2.47	1.33	1.70	2.19	3.03	N/A
stratified	physics + GBRT	1.11	1.53	0.99	1.25	1.32	1.83	1.54
split	physics + MPNN (chem)	**0.80**	**0.97**	**0.72**	**0.82**	**0.98**	**1.06**	**1.01**
split	physics	2.42	3.68	2.94	2.62	3.58	4.27	N/A
by	physics + GBRT	**1.98**	2.62	**2.48**	**2.28**	2.73	2.83	6.79
HFE	physics + MPNN (chem)	2.01	**2.35**	2.66	2.66	**2.07**	**2.64**	**6.01**
molecular	physics	1.40	2.06	1.76	1.44	1.78	2.34	N/A
scaffold	physics + GBRT	1.21	1.48	1.63	1.26	1.38	1.56	1.91
split	physics + MPNN (chem)	**1.09**	**0.82**	**1.39**	**0.96**	**0.91**	**1.00**	**1.12**

3 Supplementary Tables

Table S4: Performance of MPNN models when using **sum aggregation** instead of Set2Set aggregation. Accuracy of the physics-based hydration models with and without ensemble DNN corrections on the TEST set of 68 molecules using the **data split by HFE**. The details of the splitting procedure are found in the main text. The performance metric is RMSE relative to the experimental HFEs in kcal/mol. In bold are the best performances for the correction of each physics model. The final column titled DNN Alone shows the performance of each DNN when trained to predict HFE directly rather than predicting a correction to a physics model.

physics model	TIP3P<br>2.42	AASC<br>3.68	CHA-GB<br>2.94	GBNSR6 (ZAP9)<br>**2.62**	GBNSR6 (mbondi)<br>3.58	IGB5<br>4.27	DNN Alone<br>N/A
physics + MPNN (chem)	**1.89**	**1.99**	**2.46**	3.24	**2.13**	**2.39**	4.65
physics + MPNN (physics)	2.19	2.19	2.51	2.84	2.98	2.71	5.55
physics + MPNN (all)	1.92	2.27	2.52	3.38	2.21	2.53	**4.53**

Table S5: Performance of the physics-based hydration models with and without DNN ensemble corrections on the TEST set of 80 molecules using the **random stratified data split** without filtering out high-uncertainty experimental values. The performance metric is RMSE relative to the experimental HFEs in kcal/mol. In bold are the best performances for the correction of each physics model.

physics model	TIP3P<br>1.44	AASC<br>2.65	CHA-GB<br>1.31	GBNSR6 (ZAP9)<br>1.85	GBNSR6 (mbondi)<br>2.30	IGB5<br>3.11	DNN Alone<br>N/A
physics + GraphConv	0.99	1.53	0.95	1.43	1.31	1.68	1.47
physics + MPNN (chem)	0.89	1.17	0.95	1.10	**1.12**	1.22	1.30
physics + MPNN (physics)	0.94	1.21	0.98	**1.07**	1.14	1.25	1.43
physics + MPNN (all)	**0.82**	**1.13**	**0.91**	1.13	1.15	**1.19**	**1.25**

Table S6: Performance of the physics-based hydration models with and without DNN ensemble corrections on the TEST set of 80 molecules using the **split by HFE** without filtering out high-uncertainty experimental values as defined in the Methods section. The performance metric is RMSE relative to the experimental HFEs in kcal/mol. In bold are the best performances for the correction of each physics model.

physics model	TIP3P<br>2.47	AASC<br>3.60	CHA-GB<br>2.96	GBNSR6 (ZAP9)<br>2.60	GBNSR6 (mbondi)<br>3.44	IGB5<br>4.19	DNN Alone<br>N/A
physics + GraphConv	**1.95**	2.44	**2.39**	**2.20**	2.19	2.47	6.23
physics + MPNN (chem)	2.00	2.31	2.52	2.76	**2.07**	2.48	**5.87**
physics + MPNN (physics)	2.19	**2.27**	2.62	2.75	3.00	2.77	6.42
physics + MPNN (all)	1.99	2.47	2.52	2.61	**2.07**	**2.41**	5.92

Table S7: Performance of the physics-based hydration models with and without DNN ensemble corrections on the TEST set of 80 molecules using the **scaffold data split** without filtering out high-uncertainty experimental values. The performance metric is RMSE relative to the experimental HFEs in kcal/mol. In bold are the best performances for the correction of each physics model.

physics model	TIP3P<br>1.54	AASC<br>2.57	CHA-GB<br>2.07	GBNSR6 (ZAP9)<br>1.70	GBNSR6 (mbondi)<br>2.08	IGB5<br>2.67	DNN Alone<br>N/A
physics + GraphConv	0.90	1.36	1.40	1.26	1.20	**1.05**	1.36
physics + MPNN (chem)	1.06	1.18	1.59	1.08	1.08	1.15	**1.11**
physics + MPNN (physics)	**0.84**	1.34	**1.39**	**0.96**	**0.92**	1.12	1.31
physics + MPNN (all)	1.04	**1.07**	**1.39**	1.09	1.05	1.09	1.12

Table S8: Performance of the physics-based hydration models with and without DNN ensemble corrections on the TEST set of 76 molecules using the **random stratified data split**. The details of the splitting procedure are found in the Methods section. The performance metric is mean signed error relative to the experimental HFEs in kcal/mol. In bold are the best performances for the correction of each physics model.

physics model	TIP3P<br>0.18	AASC<br>-0.28	CHA-GB<br>-0.08	GBNSR6 (ZAP9)<br>-0.10	GBNSR6 (mbondi)<br>-0.71	IGB5<br>-1.51	DNN Alone<br>N/A
physics + GraphConv	-0.35	-0.13	-0.22	-0.27	-0.28	-0.30	0.36
physics + MPNN (chem)	-0.13	0.03	-0.03	-0.10	**-0.08**	0.05	0.10
physics + MPNN (physics)	-0.15	-0.05	0.19	-0.07	-0.18	-0.02	**-0.01**
physics + MPNN (all)	**-0.12**	**0.02**	**-0.01**	**-0.04**	-0.15	**0.00**	0.11

Table S9: Accuracy of the physics-based hydration models with and without ensemble DNN corrections on the TEST set of 68 molecules using the **data split by HFE**. The details of the splitting procedure are found in the Methods section. The accuracy metric is mean signed error relative to the experimental HFEs in kcal/mol. In bold are the best accuracies for the correction of each physics model.

physics model	TIP3P<br>**0.25**	AASC<br>-1.64	CHA-GB<br>**-0.20**	GBNSR6 (ZAP9)<br>**0.16**	GBNSR6 (mbondi)<br>-1.44	IGB5<br>-2.36	DNN Alone<br>N/A
physics + GraphConv	0.46	-0.25	0.36	0.84	-0.27	**-0.21**	5.40
physics + MPNN (chem)	0.95	**0.07**	0.90	1.29	0.35	0.83	**4.89**
physics + MPNN (physics)	0.95	0.60	1.07	1.60	1.04	1.19	5.58
physics + MPNN (all)	0.99	0.25	0.74	1.23	**0.34**	0.86	4.90

Table S10: Accuracy of the physics-based hydration models with and without ensemble DNN corrections on the TEST set of 76 molecules using the **scaffold split**. The details of the splitting procedure are found in the Methods section. The accuracy metric is mean signed error relative to the experimental HFEs in kcal/mol. In bold are the best accuracies for the correction of each physics model.

physics model	TIP3P<br>0.32	AASC<br>-0.08	CHA-GB<br>-0.50	GBNSR6 (ZAP9)<br>**0.00**	GBNSR6 (mbondi)<br>-0.54	IGB5<br>-1.30	DNN Alone<br>N/A
physics + GraphConv	-0.15	0.12	**-0.22**	-0.14	**-0.01**	**-0.01**	0.21
physics + MPNN (chem)	-0.17	-0.02	-0.29	-0.15	-0.12	-0.20	**-0.04**
physics + MPNN (physics)	**-0.07**	0.39	**-0.22**	-0.04	-0.09	0.28	-0.23
physics + MPNN (all)	-0.12	**0.01**	-0.25	-0.12	-0.07	-0.06	**-0.04**

4 Supplementary Figures

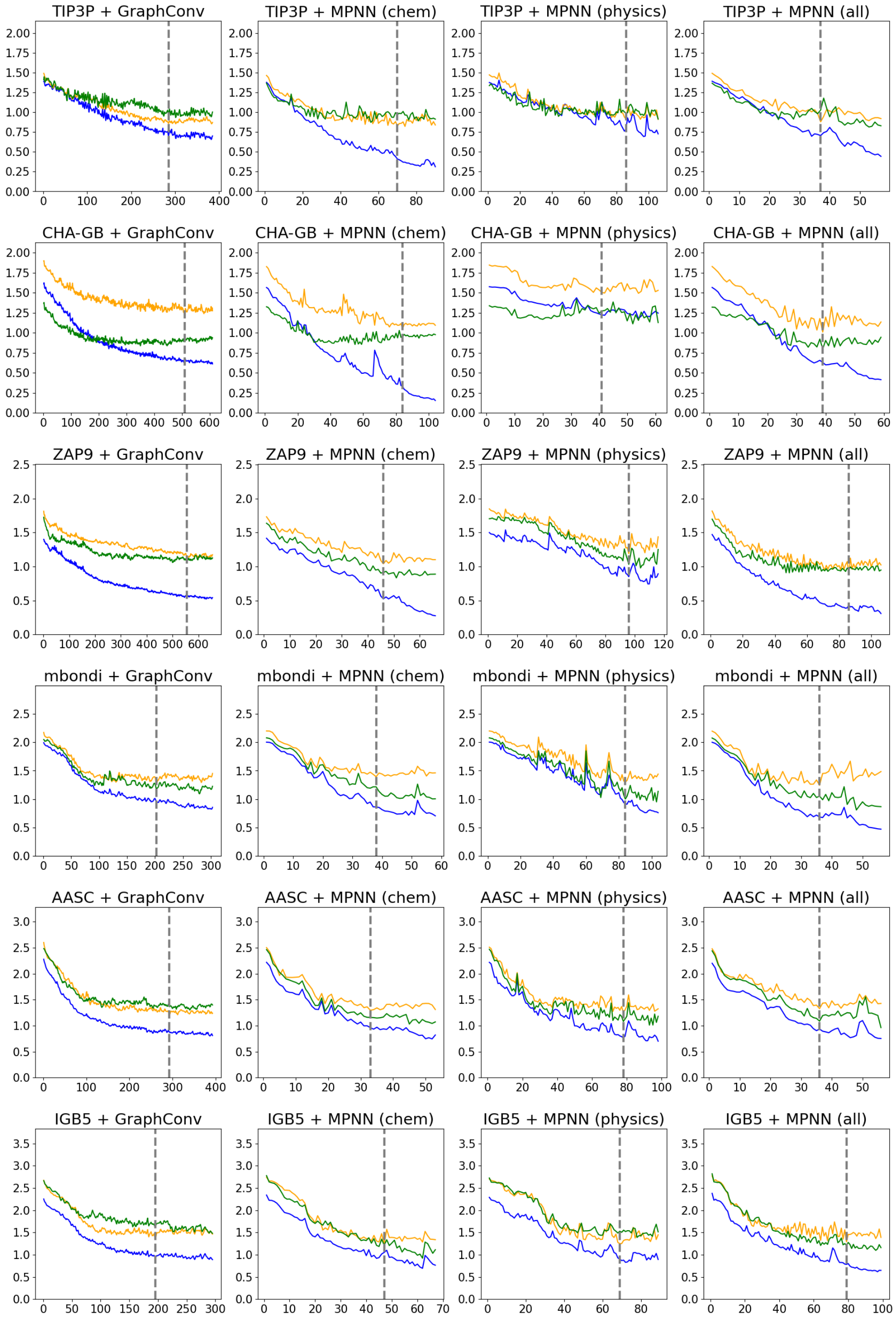

Figure S1: Convergence plots a single model out of the 20 trained for each physics model and DNN model combination using the **random stratified data split**. The y-axis for all plots is RMSE in kcal/mol and the x-axis is epochs. Green is test set, yellow is validation set, blue is train set. The dashed gray line represents the epoch selected for the final model based on early stopping criteria.

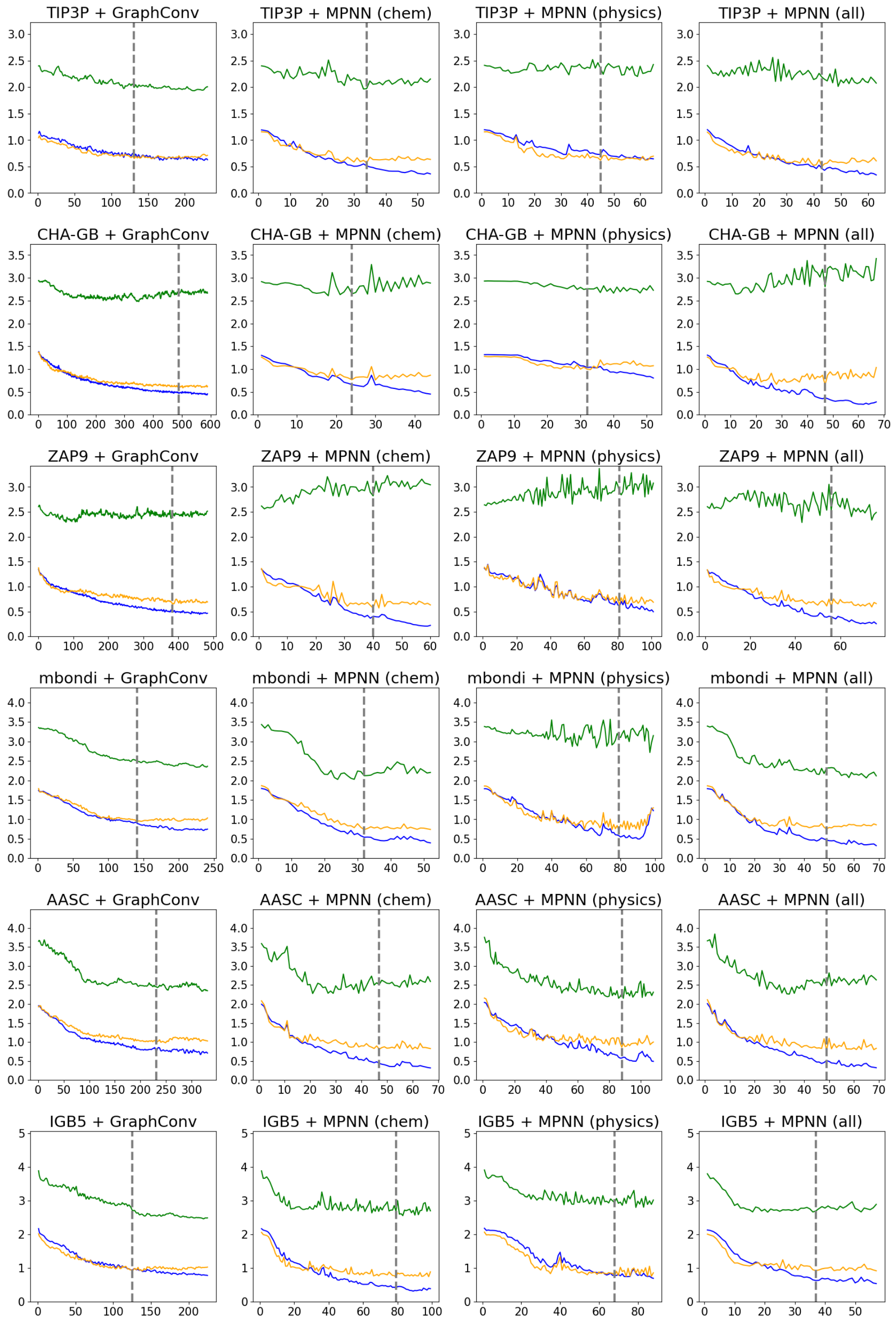

Figure S2: Convergence plots a single model out of the 20 trained for each physics model and DNN model combination using the **split by HFE**. The y-axis for all plots is RMSE in kcal/mol and the x-axis is epochs. Green is test set, yellow is validation set, blue is train set. The dashed gray line represents the epoch selected for the final model based on early stopping criteria.

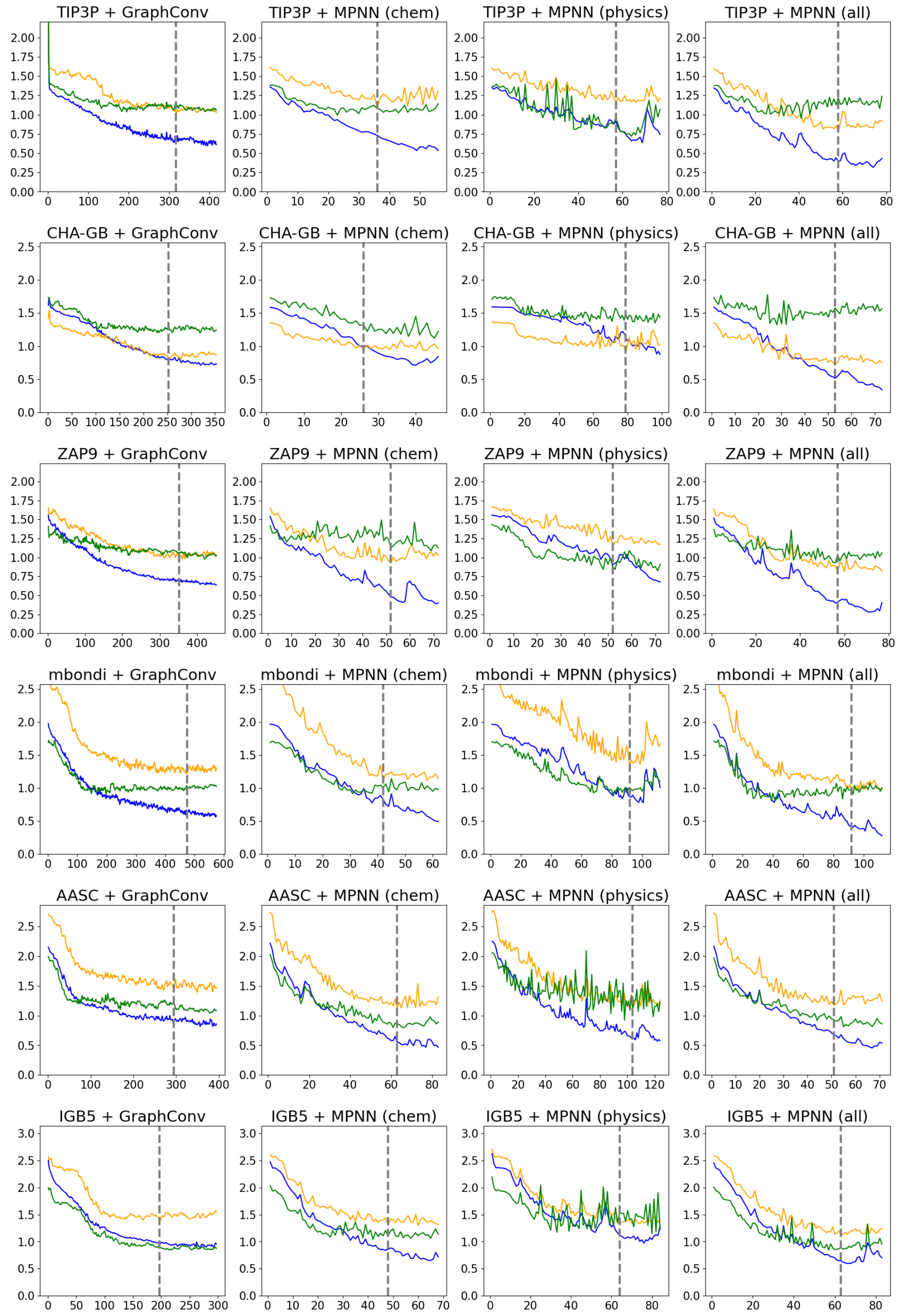

Figure S3: Convergence plots a single model out of the 20 trained for each physics model and DNN model combination using the **scaffold split**. The y-axis for all plots is RMSE in kcal/mol and the x-axis is epochs. Green is test set, yellow is validation set, blue is train set. The dashed gray line represents the epoch selected for the final model based on early stopping criteria.

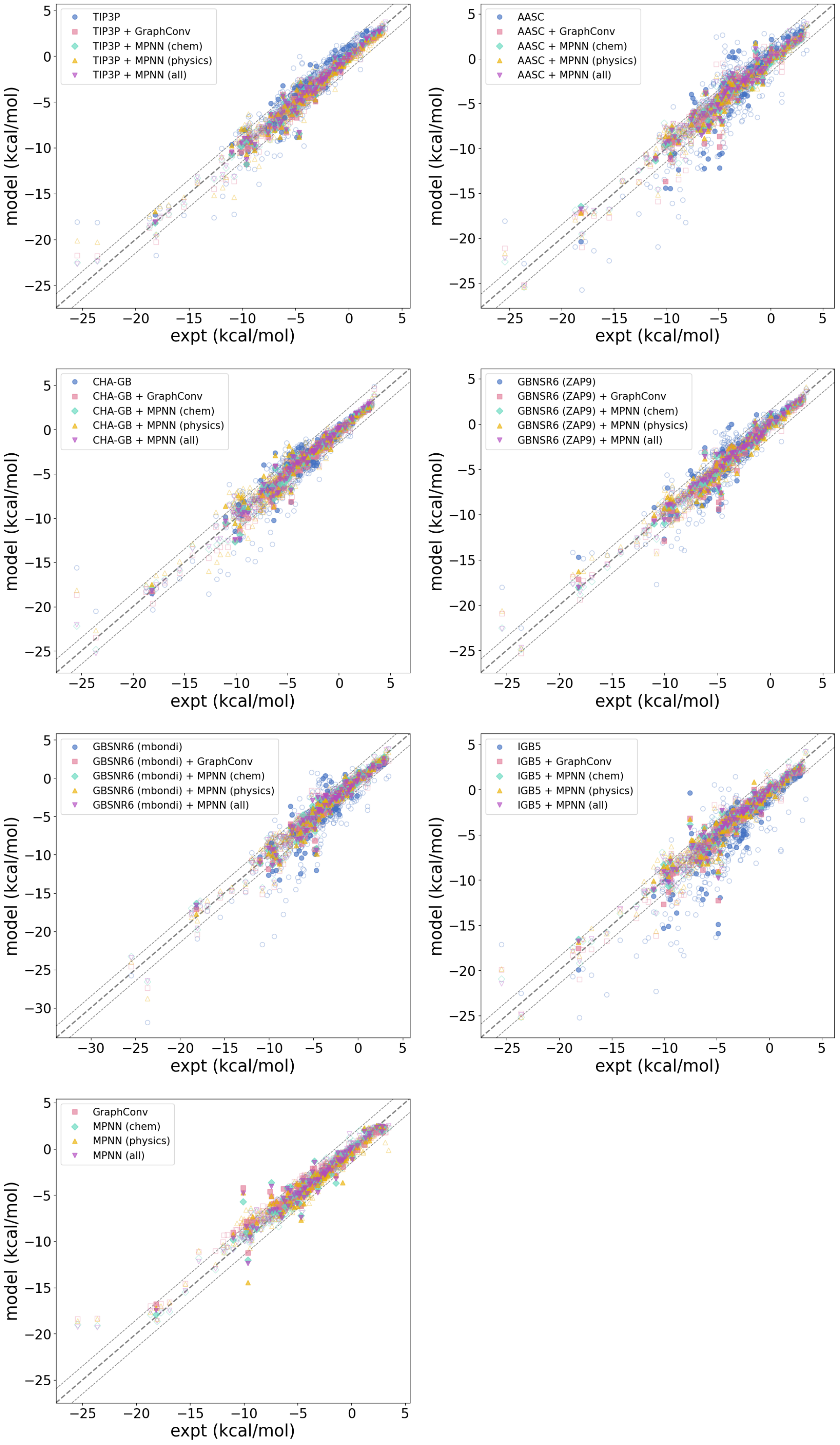

Figure S4: Scatter plots for the ensemble of the 20 trained for each physics model and DNN model combination on the **random stratified data split** test set. Test data is shown with solid points while training data is shown with hollow points. The thick dashed line indicates experiment while the thinner dashed lines show experiment ± 1.5 kcal/mol.

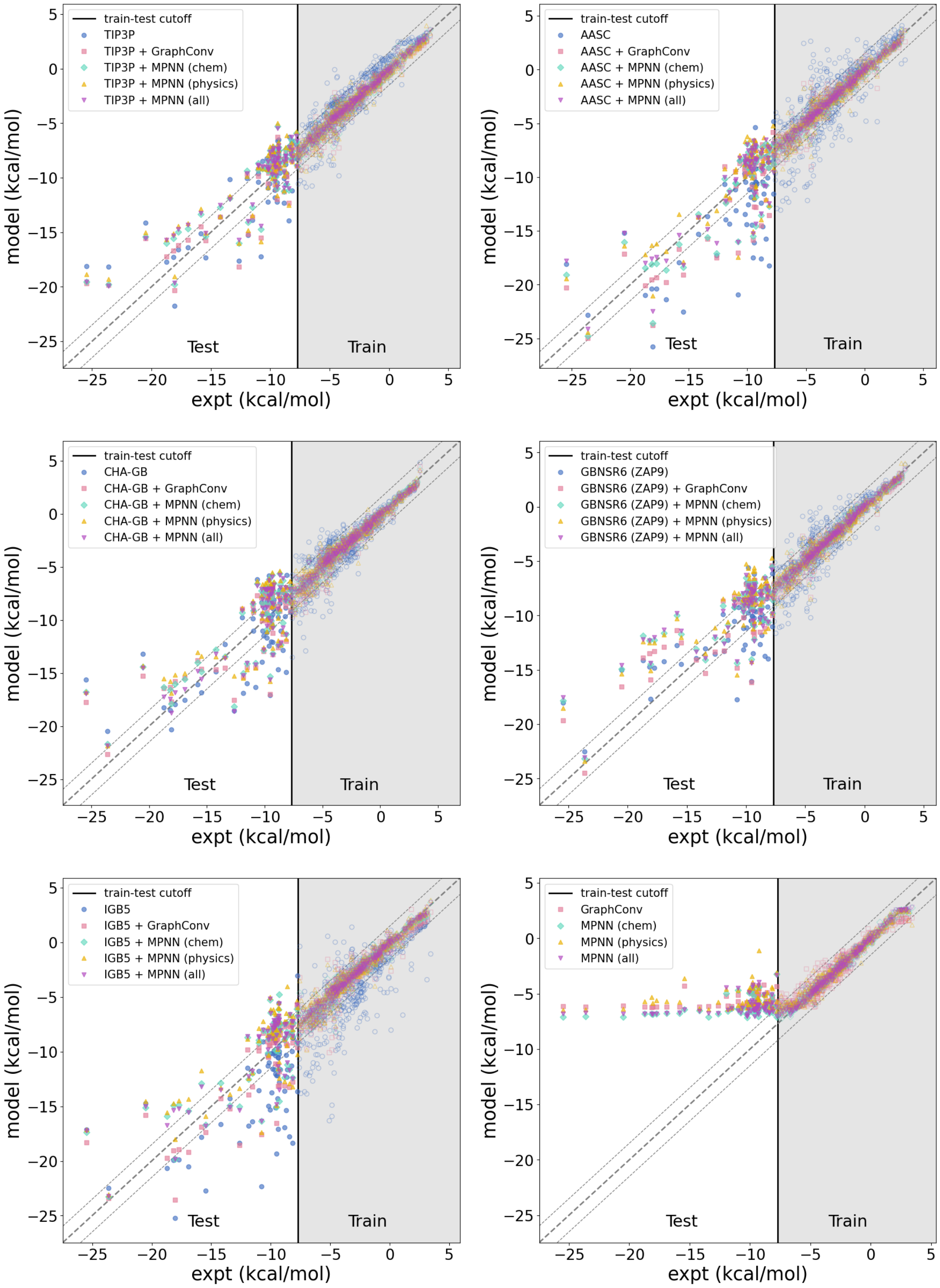

Figure S5: Scatter plots for the ensemble of the 20 trained for each physics model and DNN model combination on the **split by HFE** test set. Data to the right of the vertical line (hollow data points) are those included in the training set while data to the left of the line (solid) are used as the test set. The thick dashed line indicates experiment while the thinner dashed lines show experiment ± 1.5 kcal/mol.

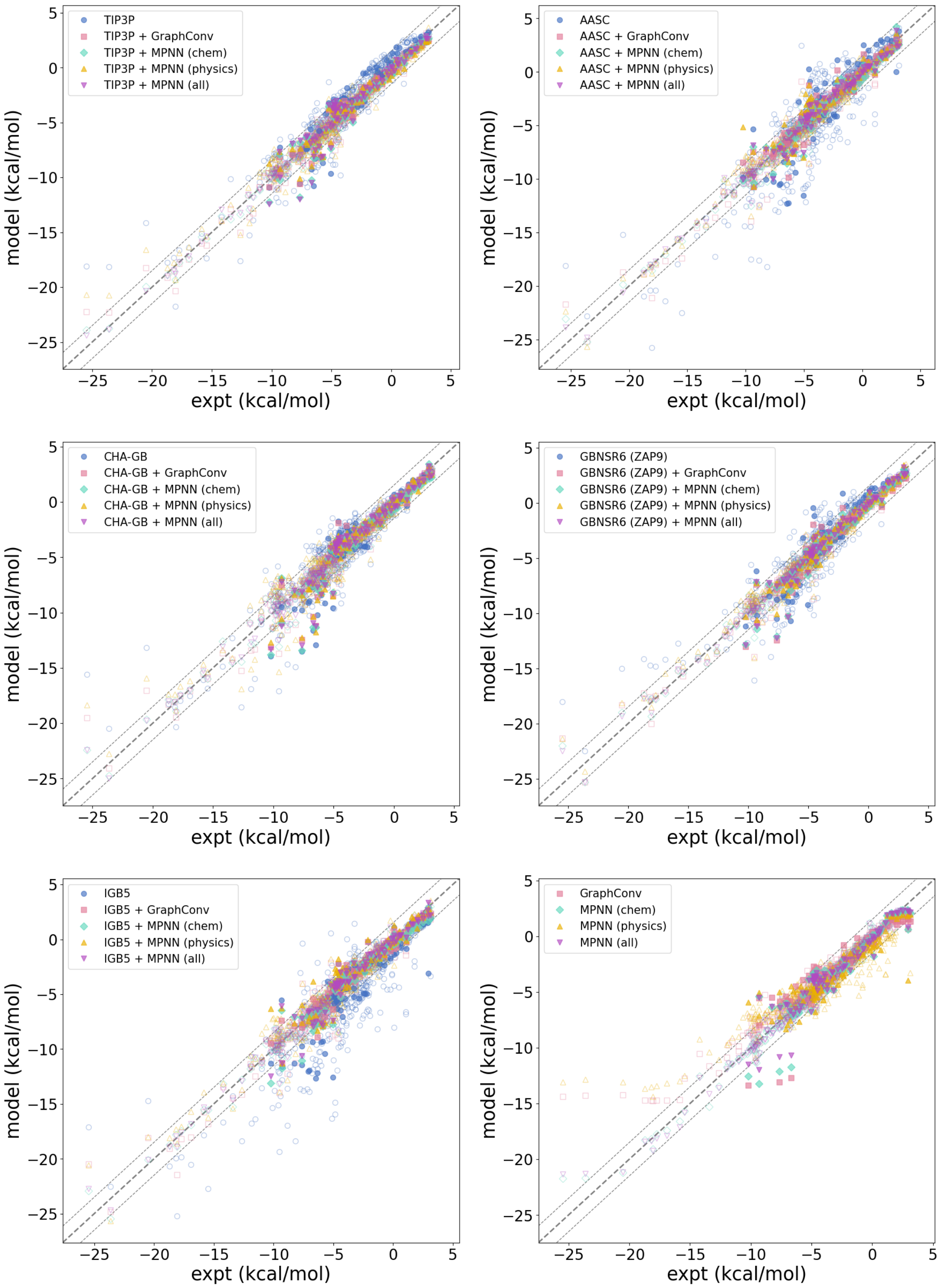

Figure S6: Scatter plots for the ensemble of the 20 trained for each physics model and DNN model combination on the **scaffold split** test set. Only molecules with ring based structures (in scaffold) are shown leaving 37 test set points (solid) and 215 training set points (hollow). The thick dashed line indicates experiment while the thinner dashed lines show experiment $\pm$ 1.5 kcal/mol.

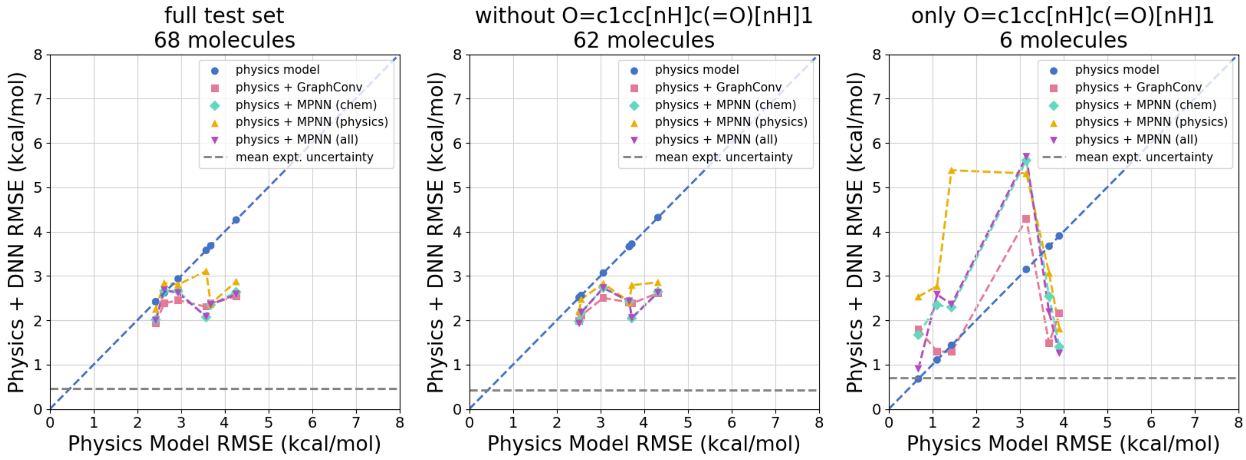

Figure S7: Extended version of Figure 4 in the main text including the results for the outlier scaffold.

5 Codes and Data Files

Source code for the training and testing of ML models, in addition to all of the saved ML models and data sets used, can be found here: github.com/Onufriev-Lab/Delta_HFE_OOD

References

(1) Jorgensen, W. L.; Chandrasekhar, J.; Madura, J. D.; Impey, R. W.; Klein, M. L. Comparison of simple potential functions for simulating liquid water. *J Chem Phys* **1983**, *79*, 926–935.

(2) Hasted, J. B. In *The Physics and Physical Chemistry of Water*; Franks, F., Ed.; Springer New York: Boston, MA, 1972; pp 255–309.

(3) Onufriev, A. V.; Saeed, I. Water models for biomolecular simulations. *Wiley Interdiscip. Rev.: Comput. Mol. Sci.* **2018**, *8*, e1347.

(4) Mobley, D. L.; Guthrie, J. P. FreeSolv: a database of experimental and calculated hydration free energies, with input files. *Journal of Computer-aided Molecular Design* **2014**, *28*, 711–720, 24928188[pmid].

(5) Mobley, D. L.; Bayly, C. I.; Cooper, M. D.; Shirts, M. R.; Dill, K. A. Small Molecule Hydration Free Energies in Explicit Solvent: An Extensive Test of Fixed-Charge Atomistic Simulations. *J. Chem. Theory Comput.* **2009**, *5*, 350–358.

(6) Onufriev, A.; Bashford, D.; Case, D. A. Exploring Protein Native States and Large-Scale Conformational Changes with a Modified Generalized Born Model. *Proteins: Struct., Funct., Bioinf.* **2004**, *55*, 383–394.

(7) Case, D. A.; Cheatham, T. E.; Darden, T.; Gohlke, H.; Luo, R.; Merz, K. M.; Onufriev, A.; Simmerling, C.; Wang, B.; Woods, R. J. The Amber Biomolecular Simulation Programs. *J. Comput. Chem.* **2005**, *26*, 1668–1688.

(8) Still, W. C.; Tempczyk, A.; Hawley, R. C.; Hendrickson, T. Semianalytical Treatment of Solvation for Molecular Mechanics and Dynamics. *J. Am. Chem. Soc.* **1990**, *112*, 6127–6129.

(9) Aguilar, B.; Shadrach, R.; Onufriev, A. V. Reducing the Secondary Structure Bias in the Generalized Born Model via R6 Effective radii. *J. Chem. Theory Comput.* **2010**, *6*, 3613–3630.

(10) Aguilar, B.; Onufriev, A. V. Efficient Computation of the Total Solvation Energy of Small Molecules via the R6 Generalized Born Model. *J. Chem. Theory Comput.* **2012**, *8*, 2404–2411.

(11) Sigalov, G.; Scheffel, P.; Onufriev, A. Incorporating Variable Dielectric Environments into the Generalized Born Model. *J. Chem. Phys.* **2005**, *122*, 094511.

(12) Roe, D. R.; Okur, A.; Wickstrom, L.; Hornak, V.; Simmerling, C. Secondary Structure Bias in Generalized Born Solvent Models: Comparison of Conformational Ensembles and Free Energy of Solvent Polarization from Explicit and Implicit Solvation. *The Journal of Physical Chemistry B* **2007**, *111*, 1846–1857, PMID: 17256983.

(13) Sigalov, G.; Scheffel, P.; Onufriev, A. Incorporating Variable Dielectric Environments Into the Generalized Born Model. *J. Chem. Phys.* **2005**, *122*, 094511.

(14) Sigalov, G.; Fenley, A.; Onufriev, A. Analytical Electrostatics for Biomolecules: Beyond the Generalized Born Approximation. *J. Chem. Phys.* **2006**, *124*, 124902.

(15) Grycuk, T. Deficiency of the Coulomb-Field Approximation in the Generalized Born Model: An Improved Formula for Born Radii Evaluation. *J. Chem. Phys.* **2003**, *119*, 4817–4826.

(16) Mongan, J.; Svrcek-Seiler, W. A.; Onufriev, A. Analysis of Integral Expressions for Effective Born Radii. *J. Chem. Phys.* **2007**, *127*, 11B608.

(17) Sanner, M. F.; Olson, A. J.; Spehner, J.-C. Reduced surface: an efficient way to compute molecular surfaces. *Biopolymers* **1996**, *38*, 305–320.

(18) Lee, B.; Richards, F. M. The interpretation of protein structures: estimation of static accessibility. *Journal of molecular biology* **1971**, *55*, 379–IN4.

(19) Forouzesh, N.; Izadi, S.; Onufriev, A. V. Grid-based surface generalized Born model for calculation of electrostatic binding free energies. *Journal of chemical information and modeling* **2017**, *57*, 2505–2513.

(20) Forouzesh, N.; Mukhopadhyay, A.; Watson, L. T.; Onufriev, A. V. Multidimensional Global Optimization and Robustness Analysis in the Context of Protein–Ligand Binding. *J. Chem. Theory Comput.* **2020**,

(21) Case, D.; Betz, R.; Cerutti, D.; T.E. Cheatham, I.; Darden, T.; Duke, R.; Giese, T.; Gohlke, H.; others AMBER 2016. *AMBER 2016, University of California, San Francisco* **2016**,

(22) Gallicchio, E.; Levy, R. M. AGBNP: An analytic implicit solvent model suitable for molecular dynamics simulations and high-resolution modeling. *Journal of computational chemistry* **2004**, *25*, 479–499.

(23) Mukhopadhyay, A.; Aguilar, B. H.; Tolokh, I. S.; Onufriev, A. V. Introducing Charge Hydration Asymmetry into the Generalized Born Model. *J. Chem. Theory Comput.* **2014**, *10*, 1788–1794.

(24) Folescu, D. E.; Onufriev, A. V. A Closed-Form, Analytical Approximation for Apparent Surface Charge and Electric Field of Molecules. *ACS Omega* **2022**, *7*, 26123–26136.

(25) Fenley, A. T.; Gordon, J. C.; Onufriev, A. An Analytical Approach to Computing Biomolecular Electrostatic Potential. I. Derivation and Analysis. *J. Chem. Phys.* **2008**, *129*, 075101.

(26) Gordon, J. C.; Fenley, A. T.; Onufriev, A. An Analytical Approach to Computing Biomolecular Electrostatic Potential. II. Validation and Applications. *J. Chem. Phys.* **2008**, *129*, 075102.

(27) Decherchi, S.; Rocchia, W. A General and Robust Ray-Casting-Based Algorithm for Triangulating Surfaces at the Nanoscale. *PLoS ONE* **2013**, *8*, e59744.

(28) Bondi, A. Van Der Waals Volumes and Radii. *J. Phys. Chem.* **1964**, *68*, 441–451.

(29) Natekin, A.; Knoll, A. Gradient boosting machines, a tutorial. *Frontiers in Neurorobotics* **2013**, *7*.

(30) Bentéjac, C.; Csörgő, A.; Martínez-Muñoz, G. A comparative analysis of gradient boosting algorithms. *Artificial Intelligence Review* **2020**, *54*, 1937–1967.

(31) Rogers, D.; Hahn, M. Extended-Connectivity Fingerprints. *Journal of Chemical Information and Modeling* **2010**, *50*, 742–754.

(32) Pedregosa, F. et al. Scikit-learn: Machine Learning in Python. *Journal of Machine Learning Research* **2011**, *12*, 2825–2830.
